# Screening for Microbial Metal-Chelating Siderophores for the Removal of Metal Ions from Solutions

**DOI:** 10.3390/microorganisms9010111

**Published:** 2021-01-05

**Authors:** Marika Hofmann, Thomas Heine, Luise Malik, Sarah Hofmann, Kristin Joffroy, Christoph Helmut Rudi Senges, Julia Elisabeth Bandow, Dirk Tischler

**Affiliations:** 1 Institute of Biosciences, TU Bergakademie Freiberg, 09599 Freiberg, Germany; heinethomas@ymail.com (T.H.); luise.malik@student.tu-freiberg.de (L.M.); hofmannsarah@aol.com (S.H.); kristin.friebel@student.tu-freiberg.de (K.J.); 2 Applied Microbiology, Faculty of Biology and Biotechnology, Ruhr-Universität Bochum, 44780 Bochum, Germany; Christoph.senges@rub.de (C.H.R.S.); julia.bandow@rub.de (J.E.B.); 3 Microbial Biotechnology, Faculty of Biology and Biotechnology, Ruhr-Universität Bochum, 44780 Bochum, Germany

**Keywords:** metallophore, screening, CAS assay, immobilization, metal binding, metal revocery

## Abstract

To guarantee the supply of critical elements in the future, the development of new technologies is essential. Siderophores have high potential in the recovery and recycling of valuable metals due to their metal-chelating properties. Using the Chrome azurol S assay, 75 bacterial strains were screened to obtain a high-yield siderophore with the ability to complex valuable critical metal ions. The siderophore production of the four selected strains *Nocardioides simplex* 3E, *Pseudomonas chlororaphis* DSM 50083, *Variovorax paradoxus* EPS, and *Rhodococcus erythropolis* B7g was optimized, resulting in significantly increased siderophore production of *N. simplex* and *R. erythropolis*. Produced siderophore amounts and velocities were highly dependent on the carbon source. The genomes of *N. simplex* and *P. chlororaphis* were sequenced. Bioinformatical analyses revealed the occurrence of an achromobactin and a pyoverdine gene cluster in *P. chlororaphis*, a heterobactin and a requichelin gene cluster in *R. erythropolis*, and a desferrioxamine gene cluster in *N. simplex.* Finally, the results of the previous metal-binding screening were validated by a proof-of-concept development for the recovery of metal ions from aqueous solutions utilizing C_18_ columns functionalized with siderophores. We demonstrated the recovery of the critical metal ions V(III), Ga(III), and In(III) from mixed metal solutions with immobilized siderophores of *N. simplex* and *R. erythropolis.*

## 1. Introduction

Due to the continuously growing world population and the increasing competition with emerging economies for resources, the industrial nations have to face new challenges in terms of securing their raw materials supply. The supply of valuable metals is significantly dependent on their occurrence, existing resources, given processing methods, and recycling possibilities. Consequently, the development of new processes that allow metal extraction from low concentrated solutions or recycling material is gaining in importance [1]. Metal-loaded wastewater discharged from manufacturing and industrial processes poses an environmental threat if not properly treated [2]. Siderophores and other natural chelators possess promising metal-binding characteristics that provide a high potential for utilization in metal extraction processes and treatment of metal-loaded waters. These low molecular weight compounds have been reported to complex different kinds of metals including valuable metals (e.g., rare earth elements (REE), actinides, Ga, In, V, Co, noble metals) and contaminating metals (e.g., Pd, Ni, Cd, U) [3,4]. Well-described siderophores are, for example, the desferrioxamines of mainly actinobacteria, yersiniabactin of proteobacteria, and pyoverdine and pyochelin of pseudomonads [5]. The affinities to different metal ions depend on the structural characteristics of the functional groups responsible for metal binding as well as cationic size and ionic charge. Although siderophores containing sulfur and nitrogen-based metal binding motifs have been reported to exhibit exceptional affinities to divalent ions like Cu(II), Zn(II), and Ni(II) [6,7,8,9], the primary functional groups are oxygen-containing hydroxamate, catecholate, and carboxylate residues. Therefore, most siderophores show a strong preference for trivalent over divalent ions [4]. 

Many siderophores have been characterized and suggested for different applications, but a major challenge for an effective application is the reusability of the chelators because of their cost-intensive production and purification. Additionally, a precise application often requires to immobilize the soluble siderophores. For the implementation of this strategy, the siderophore itself or the whole producing cell is embedded, attached, linked, or entrapped on or in a solid material. A whole cell embedment was reported with pseudomonads and rhizobacteria in Ca-alginate beads or electrospun nanofibers, where the cells were protected against toxic compounds and grown under iron-limiting conditions [10,11,12]. The successful immobilization to a sol–gel matrix has been shown for pyoverdine, parabactin, and azotobactin aiming the use as biosensors [13,14,15]. Furthermore, desferrioxamine was attached to silica films through anhydride silane or amid linker molecules [16,17] or embedded in silica-based mesocellular foam carriers [18]. Other immobilization attempts involve normal or activated forms of agarose or sepharose gels or beads [19,20,21]. Recently, the utilization of columns packed with siderophore-loaded polymeric XAD-16 resin was reported. Metals (Co, Mg, and Ni) from industrial wastewaters were removed by means of the siderophore yersiniabactin and enabled a continuous and economical process [22].

Herein, we report the search for suitable metal-chelating siderophores, which could be applied for the recovery of valuable metals, starting from a screening of over 70 strains for siderophore production as well as metal binding and ending up with siderophore-loaded solid-phase extraction columns in laboratory scale. 

## 2. Materials and Methods

All chemicals and supplements used were retrieved from Carl Roth (Karlsruhe, Germany), Sigma Aldrich (Darmstadt, Germany), Thermo Fisher (Kandel, Germany), Merck (Darmstadt, Germany).

### 2.1. Bacterial and Fungal Strains and Isolation of Siderophore-Producing Bacteria

Some of the strains used for the siderophore screening were retrieved from the German strain collection (DSMZ); others were retrieved from the strain collection of the Institute of Bioscience in Freiberg. More information about origin/references of each strain can be found in Table 1 and Table S1. Furthermore, bacteria were isolated from a heap in Neuhilbersdorf (strains H1–H3, 50°55′07.1″N 12°22′19.2″E, 2016/12/13), the sludge inside the mine drainage Roter Graben in Tuttendorf (strains RGS1–RGS4, 50°56′24.1″N 13°22′19.6″E, 2017/01/08) and wet soil next to Roter Graben (strain RGB, 2017/01/08). 1 g soil was suspended in 50 mL saline (0.9% NaCl solution) and incubated at room temperature over night while shaking. Thereafter, 100 µL were plated onto CAS (Chrome azurol S) agar plates containing 100 mg/L cycloheximide. Colonies with big yellow halos were picked and transferred to a fresh plate in order to gain single colonies. If necessary, this procedure was repeated several times. Isolates obtained were stored as glycerol stocks for subsequent work. 

### 2.2. Cultivation and Siderophore Production

Cells were cultivated in LB medium or a microorganism-specific medium. The screening experiments were carried out in culture tubes with 5 mL medium. For general siderophore production, precultures were grown three days in LB medium at 30 °C with constant shaking at 120 rpm. Subsequently, cells were harvested by centrifugation (5.000× *g*), washed three times with sterile saline, resuspended, and transferred to a minimal siderophore medium (SM) containing 12.8 g/L Na_2_HPO_4_·7 H_2_O, 3 g/L KH_2_PO_4_, 0.5 g/L NaCl, 1 g/L NH_4_Cl, 10 mL/L goodiemix, and a carbon source (e.g., 5 mM glucose or benzoate). The goodiemix solution consists of 385 mM MgSO_4_, 10 mM CaCl_2_, 0.1 mM thiamine, and 125 mL/L trace element solution (49 g/L MgCl_2_·6 H_2_O, 2 g/L CaCO_4_ 1.44 g/L ZnSO_4_·7 H_2_O, 0.85 g/L MnSO_4_ ∙ H_2_O, 0.24 g/L CuSO_4_·5 H_2_O, 0.06 g/L H_3_BO_3_, 51.3 mL/L HCl). To monitor siderophore production with the CAS assay, a low phosphate variant of the SM medium (SM-PP medium) containing only 10% of the typical phosphate concentration (1.28 g/L Na_2_HPO_4_·7 H_2_O and 0.3 g/L KH_2_PO_4_) was used to avoid interference with the assay solution. Some of the siderophore production experiments with selected strains were carried out in shaking flasks with 50 mL to 1 L medium. To investigate the light sensitivity of the siderophore/siderophore production of *V. paradoxus*, some culture tubes were lighted, while others were wrapped in aluminum foil. Lighting was realized by installing a natural light lamp (25 W, Exo Terra) in front of the incubator. All siderophore production experiments were performed using HCl (6 M) washed glassware or plastic containers to avoid iron contamination [23]. 

### 2.3. Chrome Azurol S Assay

Siderophore production was monitored using the Chrome azurol S assay as CAS agar plates as well as the liquid assay. The CAS agar plates were prepared as described in literature [24,25]. The composition of the used liquid CAS assay solution was based on the standard iron CAS assay described in 1987 with some modifications [24,26]. To screen for metal-binding ability of the siderophores, CAS assay solutions with different metals (Al, Ga, Cu, and V) were used. The preparation of the standard iron CAS assay solutions, the metal CAS assay solutions, and the assay procedure were performed as described previously [27,28,29]. In order to allow a proper determination of the siderophore concentration, control reactions of medium, buffer, or eluent were prepared in all cases. If the medium showed interference with the CAS assay, the blank sample and corresponding supernatant sample was diluted with ddH_2_O (e.g., SM medium was diluted 1:5). The calibration of the CAS assay solutions with known concentrations of DFOB (as desferrioxamine mesylate salt) allowed a semiquantitative siderophore determination in form of DFOB equivalent (DFOB_eq_) [28].

### 2.4. Siderophore Extraction and MS Analysis

Bacterial cells were removed from culture media by centrifugation (10,000× *g*, 10–25 min) followed by vacuum filtration (0.45 or 0.22 µm). The cell-free, siderophore-rich culture supernatants were adjusted to pH 6.0 and extracted using a mixture of XAD-4 and XAD-16 (each 5 g per liter culture supernatant) while stirring at 4 °C. After 20–30 h, the XAD was separated by filtration and siderophores were eluted using a defined volume of methanol. The obtained siderophore extract was concentrated by rotary evaporation at 30 °C, 180 rpm, and 90 mbar to the desired concentration. Culture supernatants and siderophore extracts were stored at 4 °C. Extracts and supernatants of *V. paradoxus* were additionally covered from light. The siderophore concentration was monitored over the time of storage. 

High-resolution mass spectrometry (HRMS) data were acquired from samples of *V. paradoxus* EPS as follows. The data were acquired using a Waters Synapt G2-S HDMS with an electrospray ionization source and a time-of-flight detector. Samples were diluted 1:10 in acetonitrile with 0.1% formic acid and injected by direct infusion with a flow of 5 µL/min. Spectra were recorded in MS^E^ positive mode over a mass range of 50 to 3000 *m*/*z* with 0.5 s per scan and leucin enkephalin injected as a reference mass every 30 s. Continuous MS^E^ spectra were recorded for 1 min with argon as collision gas and a collision energy ramp of 14–45 V. Used parameters: lockspray capillary voltage 2.5 kV, capillary voltage 2.5 kV, cone voltage 30 V, source temperature 120 °C, cone gas flow 60 L/h, flushing gas flow 550 L/h with a temperature of 150 °C. Fragment spectra were recorded in data-dependent acquisition positive mode over an energy ramp 10–25 V. The remaining parameters were used as described for MS^E^ mode.

In case of samples derived from strains *R. erythropolis* B7g and *N. simplex* 3E, the procedure was adapted, and the instrumental setup was changed. Analytes were eluted from the XAD resin by addition of 5 mL of methanol and shaking for 10 min at 40 rpm. The resin was removed by centrifugation (10,000× *g*, 10 min) and the extract diluted 1:10 in 50:50 mixture of water and acetonitrile (ACN).

Of these samples, 5 µL were injected on an ACQUITY UPLC I-Class System (Waters, Milford, Massachusetts) equipped with an ACQUITY PREMIER HSS T3 column (particle size 1.8 µm, column dimensions: 2.1 × 100 mm). A gradient with H_2_O and ACN, each with 0.1% formic acid (FA), was used with a flow rate of 0.6 mL/min (Table S4).

Data-independent MS^E^ measurements were performed with a Vion IMS QToF (Waters) with an ESI source in positive sensitivity mode. Masses in a range of 50 to 2000 *m*/*z* were detected with 0.1 s per scan and leucine enkephalin being injected as a reference mass every 5 min. Used parameters: capillary voltage 0.8 kV, sample cone voltage 40 V, source offset voltage 80 V, cone gas flow 50 L/h, desolvation gas flow 1000 L/h, source temperature 150 °C, desolvation temperature 550 °C, collision gas N_2_, collision low energy 6 V, collision high energy ramp 28–60 V. Putative siderophores were identified through matching of parent masses and comparison of fragment spectra to in silico fragmentation.

### 2.5. Screening and Optimization

The siderophore production of >70 different strains was screened, including known microorganisms and soil isolates. The strains were grown in LB medium for three days. Cells were washed and transferred to reaction tubes with 5 mL SM-PP containing 20 mM glucose. Samples were taken after three, five, and nine days and siderophore production was measured in the supernatant using the liquid CAS assay. Some strains were further tested with CAS assay solutions containing Al, Ga, Cu, or V instead of Fe. After the selection of four interesting bacteria, their siderophore production was optimized and scaled up to 100 mL or 1 L. The bacteria were grown on different carbon sources (5 mM glucose, 5 mM benzoate, 5 mM succinate, and/or 2.5 mM *n*-hexadecane) and supplied with casamino acids in different concentrations (0%, 0.2%, 0.5%, and 1.0%, and in case of *R. erythropolis*, additionally 1.5% and 2.0%). Samples were taken frequently over a period of up to 21 days.

### 2.6. Immobilization Procedure and Application to Metal-Loaded Solutions

For immobilization and metal-binding experiments, cells from LB precultures of *R. erythropolis* B7g, *V. paradoxus* EPS, *N. simplex* 3E, and *P. chlororaphis* DSM 50083 were washed and transferred 1:2 or 1:5 to 1 L SM-PP. 5 mL benzoate was added as carbon source for *V. paradoxus*, *P. chlororaphis* and *N. simplex*. *R. erythropolis* was additionally supplied with 0.2% casamino acids. In the case of *R. erythropolis* B7g, cells were cultivated with 20 mM glucose, 2.5 mM *n*-hexadecane, and 0.1% casamino acids. Siderophore concentration was monitored with the standard CAS assay. Siderophore-containing culture supernatants were separated from cells by centrifugation, adjusted to pH 2.0 using HCl, and applied to preconditioned C_18_ solid-phase extraction columns (100 mg, HyperSep^TM^, Thermo Scientific) with 250 mbar via a Biotage^®^ VacMaster (Uppsala, Schweden) (Figure S1). Depending on the siderophore concentration and the binding capacity of the column material identified in previous studies, 40 mL (*R. erythropolis*, *P. chlororaphis*) or 30 mL (*N. simplex*) culture supernatant were loaded onto the column. The siderophore concentration in the flow-through was detected to calculate the amount of siderophore attached to the column. Siderophore-containing flow-through was repetitively loaded to the columns up to four times followed by a washing step with ddH_2_O acidified with HCl to pH 2.0. The multiple loading ensured later binding of passed-through siderophore to the binding side, which were still idle after first applications. Moreover, the repetitive loading created a better distribution of the substance through the whole column. The metal-binding ability of the immobilized siderophore was examined using metal solutions of FeCl_3_∙6 H_2_O, Ga_2_ (SO_4_)_3_∙H_2_O, AlCl_3_∙6 H_2_O, CrCl_3_∙6 H_2_O, NdCl_3_∙H_2_O, CeCl_3_∙H_2_O, VCl_3_, InCl_3_, and LaCl_3_∙7 H_2_O in different concentrations and compositions. Therefore, the metal solutions were applied to the siderophore-loaded columns, incubated for 30 min at room temperature and eluted. Unloaded columns were treated the same way to function as blank samples. The flow-through was collected, a sample adjusted to 1% nitric acid and analyzed by inductively coupled plasma mass spectrometry (ICP-MS, xseries 2, Thermo Scientifc, Dreieich, Germany) [30]. All experiments were done in duplicate. A summarizing and visualizing graphic of the workflow can be found in Figure S2.

### 2.7. Genome Sequencing and Siderophore Gene Cluster 

The genome of *R. erythropolis* B7g and *V. paradoxus* EPS were published previously [31,32]. The genomic DNA of *N. simplex* 3E and the type strain *P. chlororaphis* DSM 50083 were isolated using an adapted phenol-chloroform method as described previously [31]. The preparation of the library, genome sequencing, assembly annotation, and analysis were done as described elsewhere [31,33]. 

To identify the siderophore biosynthesis gene clusters, annotation was performed using antiSMASH 5.0 [34,35] and RAST 2.0 [36,37,38]. The amino acid sequences of some key enzymes were used as queries for BLAST searches within the genome.

## 3. Results

### 3.1. Siderophore Screening 

The siderophore production and metal-binding ability of several bacterial and fungal strains were examined in order to identify good siderophore producers (≥100 µM DFOB_eq_), which can be applied to treat metal-loaded solutions. In a first screening, 72 strains were tested on solid CAS agar plates and/or with the liquid standard CAS assay for their siderophore production (Table 1 and Table S1). Except for *Oerskovia* sp. EDN3, *Sphingopyxis panaciterrulae* DSM 25122, and *Sphingopyxis italica* DSM 25299, all strains showed a positive reaction to at least one of the tests. However, the produced amount of siderophore (DFOB_eq_) detected with the liquid standard CAS assay varied from 10 µM (*Pseudomonas* sp. RGS2) to 600 µM (*P. alliaceus* DSM 813). 22 strains produced ≥100 µM siderophore (DFOB_eq_) under the standard assay conditions, 12 strains more than 250 µM. After the first screening, 25 strains were selected for the next screening round focusing on the interaction with different CAS assay variants containing the metals Al, Ga, Cu, or V instead of iron. The results are shown in Table 1. For better comparability between the strains and their different levels of siderophore production, the concentration of DFOB_eq_ of the metal CAS experiments (Me-CAS) were divided by the concentration of DFOB_eq_ of the standard Fe-CAS solution (DFOB_eq_(Me-CAS)/DFOB_eq_(Fe-CAS)) and symbolized. Three pluses (+++) indicate a better removal of the metal ion from the Me-CAS complex than from the Fe-CAS complex. The reactions with the different CAS assay solutions were diverse among the tested stains. While the siderophores of *A. aurescens*, *R. corynebacterioides*, and *T. agreste* showed no noteworthy metal removal from the Me-CAS complexes, siderophores of *Aspergillus niger*, the tested *Pseudomonas* strains, *R. erythropolis* B7g, and *Sphingomonas herbicidovorans* seem to complex at least three of the four tested metals. Furthermore, the siderophores of rhodococci showed good results with the V-CAS assay, but in most cases, comparably less interaction with the other Me-CAS solutions. 

Four strains were selected for further investigations: *R. erythropolis* B7g, *N. simplex* 3E, *P. chlororaphis*, and *V. paradoxus* EPS produced good or very good amounts of siderophores (compared with Table 1) under assay conditions and showed different metal interaction profiles. Additionally, the strains showed good homogeneous growth and were easy to handle, which were criteria for later applications. The strains belong to different genera and produce different types of siderophores, which increased the chance to find a suitable siderophore for the attachment to solid-phase extraction columns. Next to the aforementioned strains, the bacteria *T. agreste* DSM 44070, *G. rubripertincta* CWB2, and *R. erythropolis* S43 were investigated separately and the results were published elsewhere [18,29,48,49].

### 3.2. Genome Sequencing and Prediction of the Produced Siderophore 

The structures of the siderophores produced by the selected bacterial strains were not identified before. However, siderophores of representatives of pseudomonads, rhodococci, *Nocardioides* sp., and *Variovorax* sp., and the corresponding siderophore biosynthesis gene clusters are well known. The comparison of the siderophore gene clusters of our selected strains to gene clusters of biochemically and structurally described siderophores enables the estimation of the produced siderophores and their basic characteristics without the necessity of extensive purification and structure determination. 

Using RAST and antiSMASH for annotation, we were able to identify two putative siderophore biosynthesis gene clusters in the genome of *R. erythropolis* B7g (Figure S3). Both of them exhibit strong homology to gene clusters known from other rhodococci, especially strain *R. erythropolis* PR4. The first one is identified as a heterobactin biosynthesis cluster, while the respective CDS provide an identity of 96% to 100% on protein level to those from strain PR4 [50]. Heterobactins are mixed-type catecholate-hydroxamate siderophores. The second gene cluster shows homology to previously described requichelin clusters (sequence id. 95% to 100%), whose siderophore is similar to erythrochelin and foroxymithine. However, the respective siderophore has not been characterized in detail so far [50,51]. In order to verify the prediction from the genome strain, B7g was cultivated in SM-PP medium comprising 20 mM glucose, 2.5 mM *n*-hexadecane and 1% casamino acids (which was a result from the below shown optimization of siderophore production). Three biological replicates were cultivated (for 4 days) and yielded 181 to 260 µM DFOB_eq_ prior or 35 to 55 µM DFOB_eq_ after XAD extraction in culture broth, respectively. Hence, most of the siderophores were successfully extracted. Thereof, the siderophore was eluted and analyzed by means of HRMS in order to identify the main siderophore component. The results clearly demonstrated the presence of heterobactin B and apo-heterobactin S2 according to the measured *m*/*z* values 438.197 and 694.212 Da [M+H]^+^, respectively (Table S6). Those fit to the theoretical value and the fragmentation supported this annotation.

Representatives of the genus *Variovorax* have been described to produce the four carboxylate-hydroxamate siderophores variochelin, imaqobactin, variobactin, and vacidobactin and some derivates [52,53,54]. The putative siderophore gene cluster and biosynthetic modules of *V. paradoxus* EPS were identified a few years ago (Figure S4). This bioinformatic analysis indicated that structural features (such as *N*-hydroxamate moieties) known from other *Variovorax* siderophores are likely to be present in the siderophore of strain EPS [53]. Compared to the gene clusters of variobactin- and vacidobactin- producing bacteria, the siderophore gene cluster of *V. paradoxus* EPS differs in size and gene organization, indicating a larger siderophore. To prove the suggestion, we isolated the siderophore and analyzed it using mass spectrometry. The analysis showed two main signals indicating two molecules with a measured *m*/*z* of 1161.5248 and 1176.5262 Da [M+H]^+^ (Figure S5).

Since there were no genome data available for *N. simplex* 3E and *P. chlororaphis* DSM 50083 at the beginning of our study, the genomic DNA of the two strains was isolated and sequenced to receive the necessary data. This whole genome shotgun project was deposited at DDBJ/ENA/GenBank under the accession JADCLH000000000 (*P. chlororaphis*, BioSample SAMN16418570) and JADCLI000000000 (*N. simplex*, BioSample SAMN16418572). The versions described in this paper are version JADCLH010000000 and JADCLI010000000, respectively. Recently, a complete genome sequence of *P. chlororaphis* DSM 50083 was uploaded to Genbank under the accession number CP027712.1. The authors used another sequencing method, and no publication is available yet. The results of both sequencing experiments are compared in Table S2. 

A genome coverage of 210x for strain *Nocardioides simplex* 3E and 407x for strain *Pseudomonas chlororaphis* DSM 50083 was obtained, respectively. The results of the genome sequencing are summarized in Table 2. For isolate 3E, 83 contigs (≥3000 bp) were identified that cover about 5.4 Mbp with an average G + C content of 72% (N50 = 102286; N75 = 61207; L50 = 18; L75 = 36). For the strain DSM 50083, the assembly resulted in 35 contigs (≥3000 bp) covering about 6.8 Mbp with an average G + C content of 63% (N50 = 404628; N75 = 237024; L50 = 6; L75 = 12). A 16S rDNA-based phylogenetic analysis was implemented for both isolates in order to verify the closest relatives (Figures S6 and S7). *Pseudomonas chlororaphis* DSM 50083 clusters, as expected, together with the other *chlororaphis* strains (Figure S6).

Interestingly, *Nocardioides simplex* 3E does not form a branch with other simplex strains (Figure S7). To our knowledge, strain 3E has been assigned to the species simplex solely by morphological, physiological, and chemotaxonomic characters [42]; this might be a hint that a closer look at the classification is required. 

The biological subsystem distribution of the annotated genes based on RAST can be found in Table 3 [37]. A subsystem coverage of 38% and 53% was achieved for *N. simplex* 3E and *P. chlororaphis* DSM 50083, respectively. Herein, 1.0% of the genes of the strain 3E and 2.9% of the genes of strain DSM 50083 are supposed to be related to iron acquisition and metabolism. For specification, a genome analysis was performed via antiSMASH to identify siderophore biosynthesis cluster [35]. 

For *P. chlororaphis* DSM 50083, 14 secondary metabolite clusters were identified. Three of those putatively contain siderophore related genes (cluster 4, 10, 13; Table 4). However, due to the fragmentation of the genome and inaccuracies of the in silico annotation, a manual curation was required. Therewith, gene clusters for the biosynthesis of achromobactin as well as pyoverdine were identified (Figures S8 and S9). The latter finding is supported by the fact that strain DSM 50083 produces a fluorescent siderophore (data not shown). While the achromobactin cluster is highly similar to the cluster of *P. syringae* B728a (Figure S8), the pyoverdine cluster shows homology to the one from *P. fluorescens* SBW25 [55]. In contrast, two of the NRPSs (Pflu 2552 and 2553) are not encoded in strain DSM 50083. However, the three NRPSs, which are required for biosynthesis of pyoverdine, are encoded [56]. As it is known, that the structure of pyoverdines is variable [57], further analysis is needed to reveal the subsequent structure of the pyoverdine of strain DSM 50083. However, in this study we were not able to effectively apply this siderophore in our column experiments and hence no more effort was put to solve the respective siderophore structure.

In 1968, an *N. simplex* strain was described to produce ferrioxamine B and the siderophore was isolated [58]. This is in accordance with the identified siderophore biosynthesis cluster (Table 5 and Figure S10), which encodes the required enzymes known to be responsible for DFOB production. The DFO gene cluster of strain 3E provides an identity of 76–88% on protein level to the gene cluster of strain VKM Ac-2033D, which has been described previously [59,60,61]. In order to verify the genomic analysis strain, 3E was cultivated in SM-PP medium containing 5 mM benzoate and 1% casamino acids. The main cultures (three replicates) showed high siderophore titers after 4 days (486 to 549 µM DFOB_eq_) and were extracted for a subsequent HRMS study. The remaining titer was less than 8 µM DFOB_eq_ in the supernatant, and thus we can state the siderophores were almost fully extracted. Herein, four major compounds were identified, namely bisucaberin, desferrioxamine B, desferrioxamine D3 and desferrioxamine E, according to the observed masses given in Table S5; the identification using observed masses is supported by the reference compounds (theoretical masses) as well as abounded fragment masses.

### 3.3. Siderophore Production

To increase the amount of produced siderophore, the previously chosen strains were tested for their siderophore production under different media supply conditions. Since the substrate is reported to have an influence on siderophore production [62,63], the cultures were grown with glucose, succinate, benzoate, or *n*-hexadecane and regularly tested with the standard CAS assay over several days. The optical density at the inoculation and at stationary stage was measured as reference for bacterial growth (Table S3). Furthermore, the effect of the supplementation with iron-free casamino acids was investigated.

**Figure 1:**
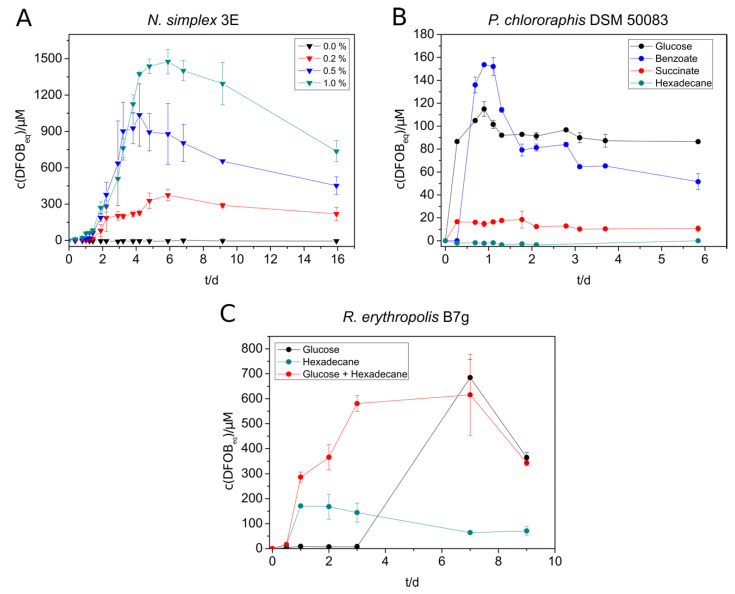
Siderophore production of selected strains under different cultivation conditions. (**A**) *N. simplex* 3E was supplied with different concentrations of casamino acids in SM-PP medium with 5 mM benzoate. (**B**) Siderophore production of *P. chlororaphis* DSM 50083 in SM-PP (siderophore medium with low phosphate content) with 5 mM succinate, benzoate, glucose, or 2.5 mM *n*-hexadecane. **C:** *R. erythropolis* B7g cultures in SM medium were grown in either 20 mM glucose, 2.5 mM *n*-hexadecane, or 20 mM glucose + 2.5 mM *n*-hexadecane as substrate. The siderophore concentration in all experiments was measured over several days using the standard CAS (Chrome Azurol S) assay. All experiments were done in triplicate (**A**) or duplicate (**B**,**C**).

*Nocardioides simplex* 3E showed the highest siderophore production with benzoate (400 µM DFOB_eq_), followed by glucose (280 µM DFOB_eq_) and succinate (180 µM DFOB_eq_) (Figure S11A). However, siderophore production in benzoate-containing cultures started later than in the cultures with other substrates. It is noteworthy that the siderophore amount increased quickly in the beginning, decreased again after reaching a maximum, and stayed constant after some days. No siderophore could be detected in *n*-hexadecane-containing cultures due to an insufficient culture growth. The produced amount of siderophore increased significantly by the addition of casamino acids. The addition of 1% yielded in concentrations up to 1500 µM DFOB_eq_ (Figure 1A). Therefore, casamino acids were added to all further siderophore producing cultures of *N. simplex*.

The results of the siderophore biosynthesis of *P. chlororaphis* with different substrates were comparable to that of *N. simplex* regarding the preferred substrates, but the maximal concentrations were significantly lower for all substrates (highest amount was 150 µM, Figure 1B). A fast decrease of the produced siderophore amount could only be observed with benzoate as substrate.

The siderophore production of *R. erythropolis* B7g cultures yielded in much higher siderophore concentrations grown on SM (Figure 1C) than on SM-PP (Figure S11B) medium due to the higher phosphate supply. The maximal produced amount (up to 680 µM DFOB_eq_) was almost three times as high as in low-phosphate medium. Small scale preliminary experiments with *R. erythropolis* showed that no noteworthy siderophore production was achieved with succinate and benzoate. Cultures grown on *n*-hexadecane showed a faster siderophore production than on glucose but a much lower siderophore concentration in SM medium. However, the simultaneous addition of both glucose and *n*-hexadecane to the culture turned out to compensate the deficits of the single substrates, leading to a fast and high siderophore production (Figure 1C). The supplementation with <1% (*m*/*v*) casamino acids and 30 mM glucose in a 1 L culture was able to increase the siderophore concentration up to 2.2 mM DFOB_eq_.

**Figure 2:**
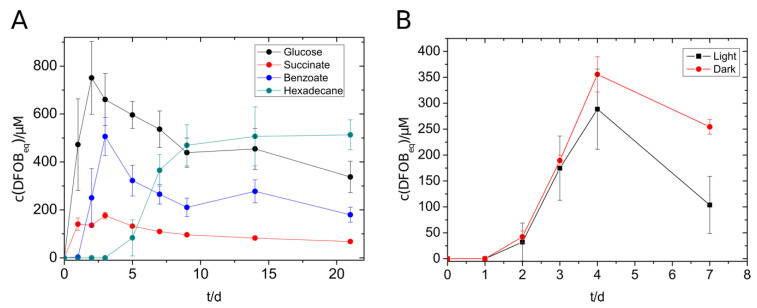
Siderophore production of *V. paradoxus* with different substrates (5 mM) (**A**) and with benzoate (5 mM) in the presence or absence of light. (**B**) Experiments were done in triplicates. The concentration of siderophores was measured using the standard CAS assay.

*V. paradoxus* EPS showed siderophore production not only with glucose, succinate, and benzoate but also with *n*-hexadecane (Figure 2A). The highest concentration of siderophore was reached with glucose (750 µM), followed by benzoate (500 µM), *n*-hexadecane (500 µM), and succinate (140 µM). The alkane-containing cultures started siderophore production not until day five, but the siderophore concentration remained at a constantly high level over the monitored period. During the siderophore production with *V. paradoxus*, we suspected that the siderophores and/or the siderophore production might be sensitive to light exposure. An experiment with lightened and darkened cultures showed a significantly increased maximal siderophore concentration in the darkened tubes (Figure 2B) supporting the previous suggestion. The formation of photoproducts needs yet to be confirmed. Nevertheless, siderophore producing cultures, siderophore-containing culture supernatants and siderophore extracts were protected from light in further experiments.

**Figure 3:**
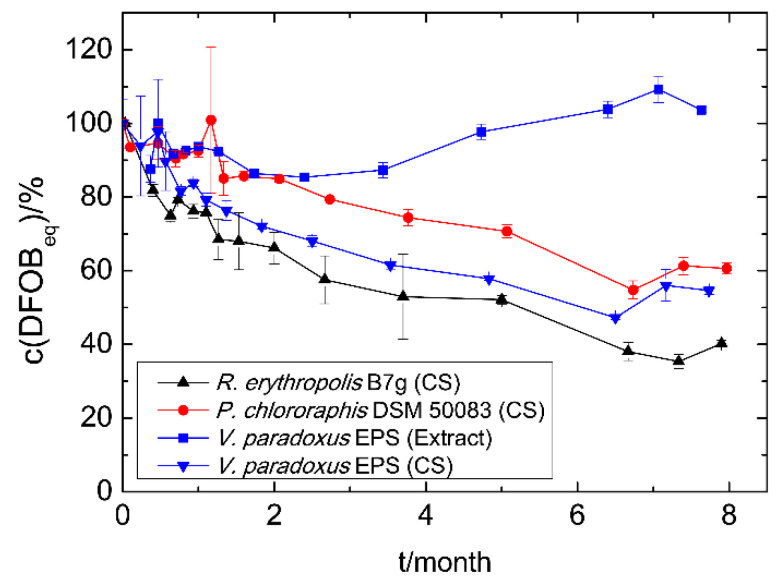
Time-dependent siderophore concentration of culture supernatants (CS) and siderophore extracts. Cell-free culture supernatants and the methanolic extract were stored at 4 °C. The concentration was measured using the CAS assay as DFOB_eq_. The initial siderophore concentration represents 100% and varied among the different strains and methods: Sid-*Re*B7g 780 µM, Sid-*Pc*DSM 110 µM, Sid-*Vp*EPS (CS) 490 µM, and Sid-*Vp*EPS (extract) 1500 µM.

The siderophores in culture supernatants of *R. erythropolis* B7g (Sid-*Re*B7g), *P. chlororaphis* DSM 50083 (Sid-*Pc*DSM), and *V. paradoxus* EPS (Sid-*Vp*EPS), which were stored at 4 °C, showed a comparable decrease in iron binding capacity over the time (Figure 3). However, the measurable concentration decrease (detected with CAS assay) appears to be slow and linear for Sid-*Pc*DSM, while 20–25% less siderophore was already detected after the first month for Sid-*Vp*EPS and Sid-*Re*B7g. Nevertheless, 40–60% of the initial CAS activity could be measured for all siderophores in culture supernatants after eight months without any addition of stabilizing supplements or other preventive actions. This indicates a good stability of the siderophores. The methanolic extract of Sid-*Vp*EPS seems to be even more stable.

### 3.4. Siderophore Attachment to Solid-Phase Extraction Columns

**Figure 4:**
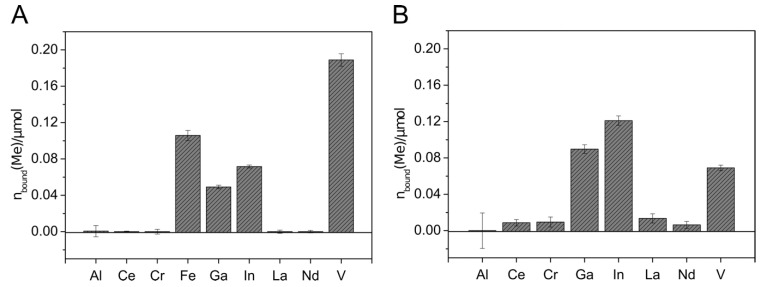
Metal binding by Sid-*Ns*3E-loaded columns. Mixed metal solutions with (**A**) and without (**B**) iron were applied to the 2.6 µmol DFOB_eq_-loaded C_18_ columns. The metal solutions contained 0.6 µmol of each different metal ion. C_18_ columns without siderophore served as blank samples. The metal concentration in the flow-through was detected using inductively coupled plasma mass spectrometry (ICP-MS xseries 2, Thermo Scientifc). The total amount of metal bound to the immobilized siderophore columns is visualized. All experiments were done in duplicate.

**Figure 5:**
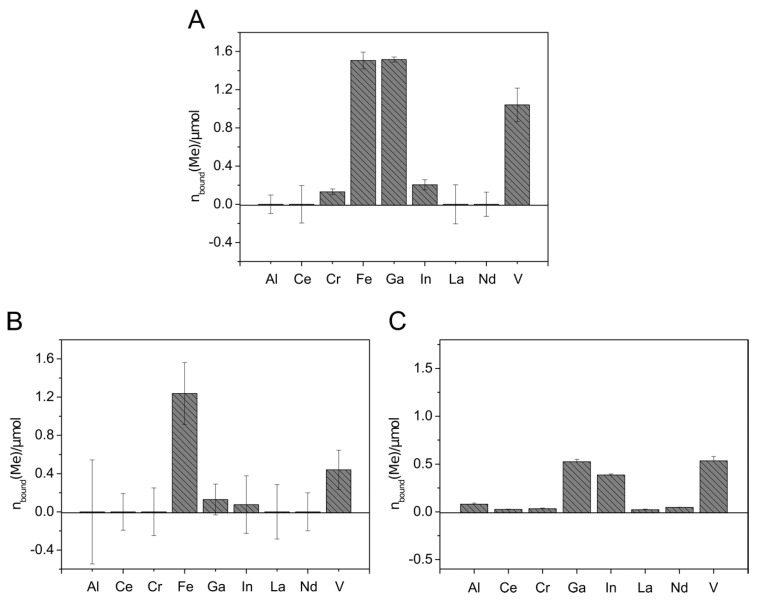
Metal binding by Sid-*Re*B7g-loaded columns. Metal solutions were applied containing either one single metal ion (14 µmol) (**A**), mixed metal ions (each 14 µmol) (**B**), or mixed metal ions (each 2 µmol) (**C**). C_18_ columns without siderophore served as blank samples. The metal concentration in the flow-through was detected using ICP-MS. The total amount of metal bound to the immobilized siderophore columns is visualized. All experiments were done in duplicates.

To remove metals from aqueous solutions, immobilization of the siderophores to solid phases simplifies the application. For that purpose, attachment of siderophores to solid-phase extraction (SPE) columns based on C_18_ and silica was investigated in a test scale (100 mg solid material) with culture supernatants containing Sid-*Pc*DSM, Sid-*Re*B7g, Sid-*Vp*EPS, or the siderophores produced by *N. simplex* 3E (Sid-*Ns*3E). The results are shown in Figure S12. Siderophores could be attached to both materials in case of Sid-*Pc*DSM and Sid-*Re*B7g; Sid-*Vp*EPS and Sid-*Ns*3E showed promising results with C_18_ columns. However, the attachment efficiency was highly pH-dependent with the best results at pH 2.0. The C_18_ material was, in general, less pH-dependent and showed a better overall attachment. Therefore, the C_18_ SPE columns were chosen for further experiments. An amount of up to 8.7 µmol Sid-*Re*B7g (13.9 µmol loaded), 3.3 µmol Sid-*Pc*DSM (5.3 µmol loaded), and 4 µmol Sid-Ns3E (13.3 µmol loaded) was immobilized to the solid material. Although the first attachment experiments revealed good results for Sid-*Pc*DSM, the siderophore proved not to be suitable for the attachment on the C_18_ columns due to a weak binding on the material. When the Sid-*Pc*DSM-columns were rinsed with several column volumes of solution, the bound siderophore was stepwise washed out over the time (data not shown). Since the siderophore production with *V. paradoxus* EPS led to reproducibility problems in larger culture volumes, the metal binding experiments with the siderophore-loaded columns were only continued with Sid-*Ns*3E and Sid-*Re*B7g. The metal binding experiments were carried out in different setups: The metals were applied as single solutions (Figure 4A), mixed solutions including Fe(III) (Figure 4A and Figure 5B), or iron-free mixed solutions (Figure 4B and Figure 5C).

The metal extraction experiment with Sid-*Ns*3E-loaded columns showed the extraction of Fe, Ga, In, and V, with higher extraction efficiency for In and Ga in the absence of Fe (Figure 5). Almost no removal could be detected with aluminum and the rare earth elements. The affinity of Sid-*Ns*3E to In(III) seems to be higher than to Ga(III) and both metals were extracted in comparable higher amounts in relation to present iron, considering the known high affinity of desferrioxamines to Fe(III). The overall extracted amount of metals was much lower with Sid-*Ns*3E than with Sid-*Re*B7g-loaded columns.

The application of metal solutions on Sid-*Re*B7g-loaded columns indicated that the immobilized siderophore(s) from *Rhodococcus* were able to complex iron and gallium to a similar extend when only a single metal was applied (Figure 5A). Additionally, indium and chromium complexed with the Sid-*Re*B7g as well as comparably high amounts of vanadium. Again, no removal could be detected for aluminum and the rare earth elements. The experiments with mixed metal solutions showed the binding of the same metals but gave an interesting insight into the binding competition. We observed a strong preference for Fe(III), leading to an amount of only 10% bound Ga(III) compared to bound Fe(III). Moreover, a stepwise application of the mixed metal solution showed that the amount of already bound gallium decreased with further addition of metal solution, while the amount of iron continued to increase (data not shown). This additionally argues for a higher affinity of Sid*-Re*B7g toward iron, as this metal seems to be able to displace gallium from the siderophore binding site. Vanadium and little amounts of indium were still complexed on the Sid-column. However, it needs to be mentioned that the high concentration of ions in the mix led to higher deviations from the metal recovery, but the overall trend as described seems valid. In a third experiment, the metals were applied in a larger, low-concentrated volume, which was pumped over the column and provides a more realistic scenario for potential applications such as wastewater treatment and metal recovery from low concentrated solutions. Additionally, a metal solution without iron was used to observe the competition in an iron-free environment. The results showed the binding of Ga, In, and V in almost similar amounts.

The immobilized siderophores could be stored at 4 °C in acidified water (pH 2.0). After 12 days (Sid-*Ns*3E) and 14 days (Sid-*Re*3E), no loss in metal binding capacity was noticed (data not shown).

## 4. Discussion

The steadily increasing demand on various metals and metalloids for our current lifestyle asks for novel strategies to enrich those elements from various streams such as low-concentrated solutions from waste streams or processing industries. Often, those nonpregnant or metal-rich solution were not identified as a source of valuable metals. However, this changed in recent years, and the various methods that might be used to extract metals or metalloids from those sources include fractional precipitation, crystallization, and solvent extraction [1,64]. All of those methods have advantages and disadvantages as reviewed in the given references. However, a critical point is that the selectivity is often low, especially in the presence of iron ions. This is problematic, as then the only concentration of a mixture but not a selective enrichment of target elements can be achieved. Here, solvent extraction is a promising route, as the formulation of novel highly selective chelators can circumvent this problem and allows the enrichment of target elements such as REE [65,66,67]. These chelators can be of synthetic or natural origin. The latter means the use of selected siderophores of microbial origin [5] and was recently reviewed and provides various possibilities [4]. Either siderophores can selectively bind iron and remove it prior further processing or they can directly enrich a target element. The progress in identifying and describing novel siderophores for selective metal enrichment achieved within this project is discussed in the following section. In order to allow easy comparison to references, we used the commercially produced and applied siderophore DFOB as reference compound.

Due to their high metal binding ability, siderophores have a great potential for application in the recovery of valuable metals. Therefore, it is of great importance to identify suitable siderophores and investigate options for efficient use. To extend the knowledge on metal binding siderophores from soil bacteria, we screened several strains for siderophore production and metal-binding ability with selected metals using different CAS assay variants. Most strains showed siderophore production to a varying extent of up to 600 µM DFOB_eq_. This is not surprising since siderophore production of different amounts is known for many soil bacteria including species of some of the investigated genera [5,10,48,68,69,70]. The detected amounts often do not reflect the full potential of siderophore production since it is dependent on carbon and energy sources, different stressors, and growth conditions of the organisms [62,63], which could not be considered and optimized individually for each strain during the screening. The limited amount of phosphate in the SM-PP medium, which was necessary to minimize the undesired interference with the CAS assay solution [24,27], led in some cases to low cell growth and consequently lower siderophore production in the test tubes. This may also be one factor that could explain the negative results with the liquid CAS assay for *S. fribergensis*, although yellow halos on the CAS agar plates clearly indicated siderophore production. When comparing the determined concentrations among the strains, it has to be considered that the assay delivers only semiquantitative results pertaining to the reaction with the used standard siderophore DFOB. Due to different molecule sizes, binding affinity, chelation chemistry, and ligand-to-cation stoichiometries of the different siderophores, the real siderophore concentration can deviate from the determined values. Considering the lack of knowledge regarding siderophore structure and chemistry of the tested subjects, the determination of the real siderophore concentrations would not be possible anyway. Therefore, the CAS assay using culture supernatants proved to be a fast and simple instrument to get an overview of the siderophore production and to identify good siderophore producers.

The optimization study of the siderophore production of four selected strains showed that the siderophore amount could be increased under optimal conditions. Notably, *R. erythropolis* produced high amounts of Sid-*Re*B7g (up to 2.2 mM DFOB_eq_). Due to the identification of two different heterobactin-like structures (Table S6), and thus various size of the siderophores, the exact yield could not be determined. However, on basis of the bioinformatical prediction assuming the production of one or more heterobactin-like siderophore and a 1:1 ligand to Fe(III) ratio of both heterobactin B/S2 and the assay standard DFOB [50,71] (deferoxamine mesylate salt M = 656 g/mol, Sigma Aldrich), the produced amount of Sid-*Re*B7g would be around 1.0–1.5 g/L, which is close to the yield of the industrial important siderophore producer *S. pilosus* [72]. The carbon source had a high influence on the siderophore amount, rapidity of the synthesis, and the decrease after reaching a maximal concentration of all four strains. Succinic acid/succinate has been reported to be the favorable carbon source for siderophore production in pseudomonads and for pyoverdine production [62,63,73]. Surprisingly, cultures of *P. chlororaphis* DSM 50083 containing succinate showed a much lower siderophore production compared to cultures grown on glucose or benzoate. A similar result was observed with *N. simplex*, although desferrioxamine siderophores are proposed to be synthesized from L-lysine and succinate units [74]. Therefore, succinate was expected to be a good substrate for desferrioxamine production in *N. simplex* as well. The desferrioxamine producing *Gordonia rubripertincta* CWB2 for instance showed a similar amount of siderophore with either glucose or succinate, although the biomass was higher in glucose-containing medium [18]. Obviously, succinate was good for siderophore production, but no good carbon source was found for *G. rubripertincta*. *V. paradoxus* was the only one of the four tested strains who yielded high amounts (up to 500 µM DFOB_eq_) of siderophore with *n*-hexadecane. The production was much slower, but it stayed on a constantly high level after several days, which might be interesting if a constant monitoring of the siderophore amount is not possible. Another interesting observation for the *V. paradoxus* cultures was the higher siderophore concentration in the absence of light than in the presence of light. Although the photolysis of the siderophores has yet to be proved, photoreactive characteristics are likely and have been described previously for siderophores from *Variovorax* species. The ferric form of variochelin from *V. boronicumulans* BAM-48 formed two photoproducts after exposition to direct sunlight, an aldehyde resulting from decarboxylation of the β-hydroxy aspartate residue as well as a second product probably resulting from a more intensive cleavage reaction [53]. Light-induced cleavage and iron reduction were also observed with the ferric complex of imaqobactin from the arctic bacterium *Variovorax* sp. RKJM285 [54]. Next to variochelin, imaqobactin, and a few other siderophores from plant-associated bacteria [75,76], photoactive siderophores have mainly been reported from marine bacteria [77,78].

After optimizing the siderophore production process, the siderophore-containing culture supernatants of *N. simplex*, *R. erythropolis*, and *P. chlororaphis* were loaded onto to small SPE columns (100 mg column material) driven by the desire to obtain immobilized siderophores. The preparation of pure siderophores is a cost-intensive process which is reflected in the high prices for commercially available siderophores (Merck: DFOE from *Streptomyces antibioticus* 9 mg 222 €, pyoverdines from *Pseudomonas fluorescens*, 1 mg 206 €, enterobactin from *Escherichia coli* 1 mg 270 €, deferriferrichrome from *Ustilago sphaerogena* 5 mg 342 €). Siderophore-rich culture supernatants, which were repeatedly pumped over the columns instead of purified siderophores, were used with the intention to reduce the costs for a potential application. The immobilization was successful for Sid-*Re*B7g from *R. erythropolis* and Sid-*Ns*3E from *N. simplex*, but no proper attachment could be achieved for Sid-*Pc*DSM from *P. chlororaphis.* The latter was slowly washed out when water or metal solution of greater volumes were applied to the siderophore-loaded columns. For Sid-*Pc*DSM, another immobilization procedure such as the embedding in sol–gel glass might deliver more promising results. Sol–gel glass (tetramethyl orthosilicate) doped with pyoverdine from *P. fluorescent* has been reported to tolerate the treatment with solutions of different pH in a flow cell without leaching of the siderophore [13].

To test the potential of the siderophore-loaded C_18_ column for metal extraction, metal solutions with different concentrations were applied, containing trivalent Al, Ga, In, Fe, V, Ce, Cr, La, and Nd ions. The removal of Fe, Ga, In, and V could be obtained with both Sid-*Re*B7g as well as Sid-*Ns*3E-loaded columns. The good binding of iron is in accordance with the literature and was expected since iron acquisition is supposed to be the main physiological role of siderophores and hydroxamate groups exhibit high affinity to Fe(III) [3,79,80,81]. However, little is known about the binding affinity of the siderophores of rhodococci besides the fact that the siderophore of *R. erythropolis* S43, a close relative of *R. erythropolis* B7g, might be involved in arsenic binding [29]. However, during our initial screening, we found that most rhodococci, which mostly produce heterobactin siderophores, are able to bind to V from the respective CAS complex; hence, it might be a hint toward a heterobactin-V complex formation to be investigated in the future (see Table 1). The results from the metal binding study with the immobilized siderophore Sid-*Re*B7g indicate an overall binding affinity of Sid-*Re*B7g of Fe > Ga/V > In > Cr with a higher extracted amount of Ga in the simple metal binding experiment but higher or equal amount of bound V with mixed metal solutions. Immobilized Sid-*Ns*3E showed comparable binding affinities but preferred In over Ga. The metal binding spectrum of the *Nocardioides* siderophore corresponds to the literature, assuming the production of desferrioxamine siderophores. Apart from iron, DFOB and DFOE have been reported to form stable complexes with Ga, Al, In [82,83,84], V [83,85], Cr [86], and REE [87]. DFOB exhibits higher binding affinities towards Ga(III) (logβ_110_ = 28.1) than In(III) (logβ_110_ = 20.1) [82]. Although the β_110_ binding constant only expresses the binding affinities of the fully deprotonated form under certain conditions (which do not necessarily match the conditions of real binding experiments), the stronger preference of Sid-*Ns*3E to In could indicate that the produced siderophore of *Nocardioides simplex* is not mainly DFOB. With the used method, the ability to bind Al(III) could be confirmed with neither Sid-*Re*B7g nor Sid-*Ns*3E in the metal binding experiment with immobilized siderophores. Since the decolorization of the Al-CAS assay indicated Al(III) complexation during the screening experiment, this is in contrast to our expectations. Al(III) shows a high similarity to Fe(III) and Ga(III) and is reported to form stable complexes with many siderophores, including desferrioxamines [82,88,89,90]. Therefore, further experiments are necessary to elucidate the Al(III) binding ability. The siderophore-loaded columns were not able to extract rare earth elements in a sufficient way. An explanation could be found in the large ionic radii of REE, making them too bulky for a sufficient coordination resulting in low binding affinities [87]. A more promising result is the high removal of vanadium from metal solutions. The formation of colored vanadium complexes has been reported for DFOB with V(V) as VO_4_^3−^ and V(IV) as VO^2+^ ion [85,91] with low binding affinities. However, the binding of V and Mo is widely known for siderophores from nitrogen-fixing bacteria, which complex these metals with exceptionally high stabilities [92,93]. The overall results confirm the potential of the immobilized siderophores for the recovery of metals such as Ga, In, and V from low concentrated waters. For instance, the recycling of Ga from wastewater of the wafer fabrication industry is a promising option to apply the developed system. Recycling ensures the future availability of this critical valuable metal, but conventional methods like solvent extraction processes [94], precipitation [95], and ion exchangers [96] failed in respect to an economic recovery due to low Ga concentrations (less than 100 mg/L) and contaminants such as Zn(II) and Ca(II) [84,97]. Another promising application could be the recovery of V from seawater, which is the second most abundant dissolved transition metal in seawater with concentrations of about 30 nM [98]. However, the proposed system of this study needs to be scaled up and tested with real water samples. Another important aspect is the recovery of the siderophore-loaded columns to create a recyclable system. The application of EDTA has demonstrated to regenerate desferrioxamines from the corresponding Ga-complexes [84] and is worth investigating with the immobilized siderophores.

In summary, we present the search for a high yield siderophore with the ability to complex valuable critical metals such as Ga, In, V, and REE, the immobilization of selected siderophores to SPE columns, and a proof-of-concept study for the siderophore-based recovery of critical metals from aqueous solutions. Although further research is necessary to generate a fully developed system, the immobilized siderophores of *R. erythropolis* and *N. simplex* were able to recover Ga, In, and V, opening doors in the recovery of critical metals.

## Figures and Tables

**Table 1:** Results of the screening of selected strains with different Chrome azurol S (CAS) assay solutions.

Strain	Origin/Reference	CAS Agar Plates	Standard Fe-CAS (DFOB_eq_)	Al-CAS	Ga-CAS	Cu-CAS	V-CAS
**Fungi**							
*Aspergillus niger* DSM 821	DSMZ	n.d.	++ (180 µM)	+++	+++	+++	+++
*Cunninghamella elegans* DSM 1908	DSMZ	n.d.	+++ (500 µM) ^b^	n.d.	++	++	+
*Penicillium griseofulvum* DSM 896	DSMZ	n.d.	+ (90 µM)	+	-	+++	+
*Petromyces alliaceus* DSM 813	DSMZ	n.d.	+++ (600 µM) ^b^	++	++	+	+
**Bacteria**							
*Arthrobacter aurescens* TC1	Local strain collection, [39]	++	+++ (280 µM)	+	+	+	+
*Gordonia rubripertincta* CWB 2 DSM 46758	Local strain collection, isolated from soil [40,41]	+ ^a^	+ (70 µM)	++	+	+++	n.d.
*Nocardioides simplex* 3E	Local strain collection, [42]	++	+++ (340 µM)	+	-	++	(+)
*Oerskovia* sp. SPF2	Local strain collection, isolated from soil [40]	+	++ (170 µM)	++	+	+	+++
*Paracoccus denitrificans* DSM 413	DSMZ	+++	+++ (400 µM)	+	+	++	++
*Pseudomonas* sp. RGS3	Soil isolate, unpublished	+++	+ (85 µM)	++	-	+++	++
*Pseudomonas* sp. RGS4	Soil isolate, unpublished	+++	++ (110 µM)	+++	-	++	++
*Pseudomonas* sp. RGB	Soil isolate, [33]	+++	++ (110 µM)	++	++	+++	+++
*Pseudomonas* sp. H1	Soil isolate, unpublished	+++	++ (140 µM)	+++	+	++	++
*Pseudomonas* sp. H3	Soil isolate, [33]	+	+ (60 µM)	+++	+	+++	++
*Pseudomonas chlororaphis* DSM 50083	DSMZ	n.d.	++ (110 µM)	+	++	+++	++
*Rhodococcus* sp. R2 (2)	Local strain collection *, [43]	+++	+++ (340 µM)	++	+	+	+++
*Rhodococcus corynebacterioides* DSM 20151	DSMZ	+	+++ (270 µM)	+	+	+	+
*Rhodococcus erythropolis* B7g	Local strain collection [31]	+++	+++ (350 µM)	++	+	+++	+++
*Rhodococcus erythropolis* S43	Local strain collection [29,44,45]	++	++ (160 µM)	+	+	+	+++
*Rhodococcus jostii* RHA1	[46]	+++	++ (100 µM)	++	+	+++	n.d.
*Rhodococcus opacus* 1CP, DSM 46757	DSMZ [47]	++	++ (150 µM)	++	+	++	n.d.
*Rhodococcus ruber* DSM 8425	DSMZ	++	++ (230 µM)	+	+	+	+++
*Sphingomonas herbicidovorans* DSM 11019	DSMZ	++	++ (110 µM)	++	+++	+++	+
*Thermocrispum agreste* DSM 44070	DSMZ	++ ^c^	+++ (300 µM) ^c^	n.d.	+	+	+
*Variovorax paradoxus* EPS	Local strain collection, P. Orwin, personal communication, [32]	++	+++ (470 µM)	+	++	+++	+

* Previously obtained from the strain collection of L. A. Golovleva (G. K. Skryabin Institute of Biochemistry and Physiology of Microorganisms, Russian Academy of Sciences, Pushchino). CAS agar plates: small halos (+), medium halos (++), big halos (+++). Different screening conditions are indicated: ^a^ low cell growth, ^b^ cultivation at room temperature, ^c^ cultivation at 45 °C. The siderophore production for the screening with liquid CAS assay was carried out in SM-PP medium with 20 mM glucose as carbon source, after cell transfer. Liquid CAS assay variants: Culture supernatants were tested and the siderophore concentrations as DFOB_eq_ were calculated as described previously [28]. 0–100 µM (+), 100–250 µM (++), >250 µM (+++). Screening results for metal binding are expressed as the ratio of DFOB_eq_(Fe-CAS):DFOB_eq_(Me-CAS). DFOB_eq_(Al-CAS):DFOB_eq_(Fe-CAS) ratio 0 to 0.5 (+), 0.51 to 1 (++), >1 (+++). DFOB_eq_(Ga-CAS):DFOB_eq_(Fe-CAS) ratio 0 to 0.3 (+), 0.31 to 1 (++), >0.6 (+++) DFOB_eq_(Cu-CAS):DFOB_eq_(Fe-CAS) ratio 0 to 0.5 (+), 0.51 to 1 (++), >1 (+++). DFOB_eq_(V-CAS):DFOB_eq_(Fe-CAS) ratio 0 to 0.5 (+), 0.51 to 1 (++), >1 (+++).

**Table 2:** Genome statistics of the genome sequenced strains.

Genome Feature	*Nocardioides simplex* 3E	*Pseudomonas chlororaphis* DSM 50083
Sum of contig length (bp)	5,365,546	6,771,389
Contigs	83 (>3000 bp)	35 (>3000 bp)
G + C content (%)	72	63
Protein coding genes	5148	6128
Average gene length (bp)	944	978
Coding percentage (%)	91	89
tRNAs	46	43
rRNAs	4	2

**Table 3:** Biological subsystem distribution of annotated genes in *N. simplex* 3E and *P. chlororaphis* DSM 50083.

	*Nocardioides simplex* 3E		*Pseudomonas chlororaphis* DSM 50083	
Subsystem Coverage	38%		53%	
Description	Value	Percent	Value	Percent
Cofactors, vitamins, prosthetic groups, pigments	366	11.9	385	8.1
Cell wall and capsule	76	2.5	232	4.9
Virulence, disease, and defense	100	3.3	153	3.2
Potassium metabolism	13	0.4	26	0.5
Miscellaneous	39	1.3	91	1.9
Phages, prophages, transposable elements, plasmids	5	0.2	37	0.8
Membrane transport	116	3.8	310	6.5
Iron acquisition and metabolism	31	1.0	137	2.9
RNA metabolism	99	3.2	223	4.7
Nucleosides and nucleotides	123	4.0	142	3.0
Protein metabolism	267	8.7	287	6.0
Cell division and cell cycle	8	0.3	33	0.7
Motility and chemotaxis	61	2.0	117	2.5
Regulation and cell signaling	46	1.5	163	3.4
Secondary metabolism	8	0.3	8	0.2
DNA metabolism	109	3.5	101	2.1
Fatty acids, lipids, and isoprenoids	296	9.6	232	4.9
Nitrogen metabolism	29	0.9	84	1.8
Dormancy and sporulation	2	0.1	3	0.1
Respiration	108	3.5	153	3.2
Stress response	114	3.7	229	4.8
Metabolism of aromatic compounds	76	2.5	161	3.4
Amino acids and derivatives	502	16.3	783	16.4
Sulfur metabolism	48	1.6	107	2.2
Phosphorus metabolism	51	1.7	83	1.7
Carbohydrates	378	12.3	493	10.3

**Table 4:** Secondary metabolite clusters identified in *P. chlororaphis* DSM 50083 with antiSMASH 5.0.

Cluster	Type	From	To	Most Similar Known Cluster	Similarity
1	Betalactone	79,159	102,356	Fengycin	13%
2	Butyrolactone	398,878	409,684	-	-
3	Hserlactone, phenazine	653,018	675,803	Streptophenazine	21%
4	NRPS	10,215	63,258	Pyoverdin	22%
5	Hserlactone	90,777	111,436	-	-
6	Bacteriocin	267,812	278,636	-	-
7	NAGGN	125,396	140,044	-	-
8	Bacteriocin	263,712	274,605	-	-
9	NRPS	371,707	421,496	Ashimides	8%
10	Siderophore	474,501	493,491	Vibrioferrin	18%
11	Other	592,690	633,775	Pyrrolnitrin	100%
12	Arylpolyene	184,548	228,168	APE Vf	40%
13	NRPS, resorcinol	122,372	205,824	Pyoverdin	31%
14	NRPS-like	1	31,154	Fragin	60%

**Table 5:** Secondary metabolite clusters identified in *N. simplex* 3E with antiSMASH 5.0.

Cluster	Type	From	To	Most Similar Known Cluster	Similarity
1	NRPS-like	27,311	69,866	-	-
2	Siderophore	37,825	50,206	Desferrioxamine	50%
3	NRPS-like	78,561	122,328	-	-

## Data Availability

The genome data presented in this study are openly available in DDBJ/ENA/GenBank at the reference under the accession JADCLH000000000 (*P. chlororaphis*, BioSample SAMN16418570) and JADCLI000000000 (*N. simplex*, BioSample SAMN16418572).

## Supplementary Materials

The following are available online at https://www.mdpi.com/2076-2607/9/1/111/s1, Table S1: Results of the screening with different CAS agar plates and liquid CAS assay, Figure S1: Experimental setup of siderophore attachment to C_18_ solid-phase extraction columns, Figure S2: Workflow of cultivation, supernatant harvest, siderophore attachment and metal binding examination in bound state, Figure S3: Organization of the biosynthesis gene cluster of *R. erythropolis* B7g, Figure S4: Organization of the biosynthesis gene cluster of *V. paradoxus* and other *Variovorax* sp., Figure S5: Mass spectrum of the siderophores of *Variovorax paradoxus* EPS, Table S2: Comparison of the genome sequences of *P. chlororaphis* DSM50083, Figure S6: Phylogenetic tree of *Pseudomonas chlororaphis* relatives., Figure S7: Phylogenetic tree of *Nocardioides simplex* relatives, Figure S8: Organization of the achromobactin biosynthesis gene clusters in *P. chlororaphis* DSM 50083 and *P. syringae* B728a, Figure S9: Organization of the pyoverdine biosynthesis gene cluster in *P. chlororaphis* DSM 50083, *P. aeruginosa* PAO1, and *P. fluorescens* SBW25, Figure S10: Organization of the biosynthesis gene cluster in *N. simplex* 3E and other strains containing desferrioxamine biosynthesis gene cluster, Table S3: Optical density at inoculation and at stationary stage as reference for bacterial growth under different growth condition, Figure S11: Siderophore production of *N. simplex* 3E and *R. erythropolis* B7g with different substrates and SM-PP medium, Figure S12: Siderophore attachment on solid-phase extraction columns, Table S4: Liquid chromatography gradient, Table S5: Siderophores detected in XAD extracts of *N. simplex* 3E, and Table S6: Siderophores detected in XAD extracts of *R. erythropolis* B7g.
