# Supplementary material for: Screening for Microbial Metal-Chelating Siderophores for the Removal of Metal Ions from Solutions
Source: Microorganisms. 2021 Jan 5;9(1):111. doi: 10.3390/microorganisms9010111 (PMC7824959; doi:10.3390/microorganisms9010111)
Supplement: Supplementary file 1 [file microorganisms-09-00111-s001.pdf]

## Supplementary Materials

**Table S1**. Results of the screening with different CAS agar plates and liquid CAS assay.

| Strain | Origin/Reference | CAS agar plates | Standard Fe-CAS (liqu.) |
|---|---|---|---|
| *Acidovorax facilis* UFZ-B530 | Local strain collection, UFZ Leipzig [1] | + | ++ (190 μM) |
| *Acinetobacter calcoaceticus* ADP1 wildtype | Local strain collection [2] L.N. Ornston | + | + |
| *Aliivibrio fischeri* DSM 507 | Local strain collection, DSMZ | +++ | n.d. |
| *Corynebacterium glutamicum* DSM 20300 | Local strain collection, DSMZ | - | + (70 μM) |
| *Cupriavidus necator* H16 DSM 428 | Local strain collection, DSMZ | ++ | n.d.[a] |
| *Escherichia coli* BL21 (DE3) | NEB GmbH | n.d. | + |
| *Gordonia* sp. CWB1 | Local strain collection, isolated from soil sample [3] | +[a] | n.d. |
| *Gordonia* sp. CWB3 | Local strain collection, isolated from soil sample [3] | +[a] | n.d. |
| *Gordonia* sp. CWB4 | Local strain collection, isolated from soil sample [3] | ++[a] | n.d. |
| *Gordonia* sp. CWB5 | Local strain collection, isolated from soil sample [3] | ++[a] | n.d. |
| *Gordonia* sp. CWB7 | Local strain collection, isolated from soil sample [3] | ++[a] | n.d. |
| *Gordonia* sp. CWB8 | Local strain collection, isolated from soil sample [3] | ++[a] | + (70 μM) |
| *Gordonia* sp. CWB10 | Local strain collection, isolated from soil sample, [3] | ++[a] | n.d. |
| *Gordonia* sp. CWB11 | Local strain collection, isolated from soil sample [3] | +[a] | n.d. |
| *Gordonia* sp. CWB16 | Local strain collection, isolated from soil sample [3] | +[a] | n.d. |
| *Gordonia* sp. CWB17 | Local strain collection, isolated from soil sample [3] | +[a] | n.d. |
| *Micrococcus luteus* DSM 12735 | DSMZ | - | + |
| *Oerskovia* sp. SPF1 | Local strain collection, isolated from soil sample [3] | - | n.d. |
| *Oerskovia* sp. EDN3 | Local strain collection, isolated from soil sample [3] | - | - |
| *Pseudomonas* sp. RGS1 | Soil isolate, unpublished | +++ | + (15 μM) |
| *Pseudomonas* sp. RGS2 | Soil isolate, unpublished | | + (10 μM) |
| *Pseudomonas* sp. H2 | Soil isolate, unpublished | +++ | + (25 μM) |
| *Pseudomonas fluorescens* DSM 14292 | DSMZ | n.d. | + (50 μM) |
| *Pseudomonas putida* DSM 12735 | DSMZ | n.d. | + (30 μM) |
| *Rhodococcus* sp. CWB12 | Local strain collection, isolated from soil sample [3] | +++ | + |
| *Rhodococcus* sp. CWB13 | Local strain collection, isolated from soil sample [3] | + | n.d. |
| *Rhodococcus* sp. CWB15 | Local strain collection, isolated from soil sample [3] | + | n.d. |
| *Rhodococcus opacus* DSM 43943 | DSMZ | ++ | n.d. |
| *Rhodococcus* sp. 3 L | Local strain collection*, Isolated from contaminated soil sample [4] | ++ | n.d. |
| *Rhodococcus* sp. 4 L | Local strain collection*, Isolated from contaminated soil sample [4] | + | n.d. |
| *Rhodococcus opacus* MR11 DSM 43205 | DSMZ | + | n.d. |

| Strain | Origin/Reference | CAS agar plates | Standard Fe-CAS (liqu.) |
|---|---|---|---|
| *Rhodococcus* sp. PM1 | Local strain collection [4] | ++ | n.d. |
| *Rhodococcus* sp. US-B1 | Local strain collection, Frauenhofer-Gesellschaft Stuttgart [4] | + | n.d. |
| *Rhodococcus erythropolis* BD2.101 | Local strain collection [5] | ++ | n.d. |
| *Rhodococcus erythropolis* DSM 43066 | DSMZ | + | ++ (100 µM) |
| *Rhodococcus opacus 557* | Local strain collection*[4] | ++ | n.d. |
| *Rhodococcus rhodnii* 135 | Local strain collection*[4] | + | n.d. |
| *Rhodococcus rhodochrous* 172 | Local strain collection* [4] | +++ | - |
| *Rhodococcus rhodochrous S5* DSM 6697 | DSMZ | + | + (35 µM) |
| *Rhodococcus rhodochrous 89* | Local strain collection* [4] | +[a] | n.d. |
| *Rhodococcus zopfii* DSM 44108 | DSMZ | + | + (40 µM) |
| *Sphingopyxis bauzanensis* DSM 22271 | DSMZ | n.d. | + (50 µM) |
| *Sphingopyxis chilensis* DSM 14889 | DSMZ | n.d. | + (50 µM) |
| *Sphingopyxis fribergensis* Kp5.2 DSM 28731 | Local strain collection, isolated from soil sample [6,7] | ++ | - |
| *Sphingopyxis italica* DSM 25299 | DSMZ | n.d. | - |
| *Sphingopyxis panaciterrulae* DSM 25122 | DSMZ | n.d. | - |
| *Xanthobacter* sp. DSM 6696 | DSMZ | - | + |

* previously obtained from the strain collection of L. A. Golovleva (G. K. Skryabin Institute of Biochemistry and Physiology of Microorganisms, Russian Academy of Sciences, Pushchino). CAS agar plates: no halos (-), small halos (+), medium halos (++), big halos (+++). [a] low cell growth. The siderophore production for the screening with liquid CAS assay was carried out in SM-PP medium with 20 mM glucose as carbon source, after pre-grown cell transfer. Liquid CAS assay variants: Culture supernatants were tested and the siderophore concentrations as $DFOB_{eq}$ were calculated as described previously [8] 0–100 µM (+), 100–250 µM (++), > 250 µM (+++).

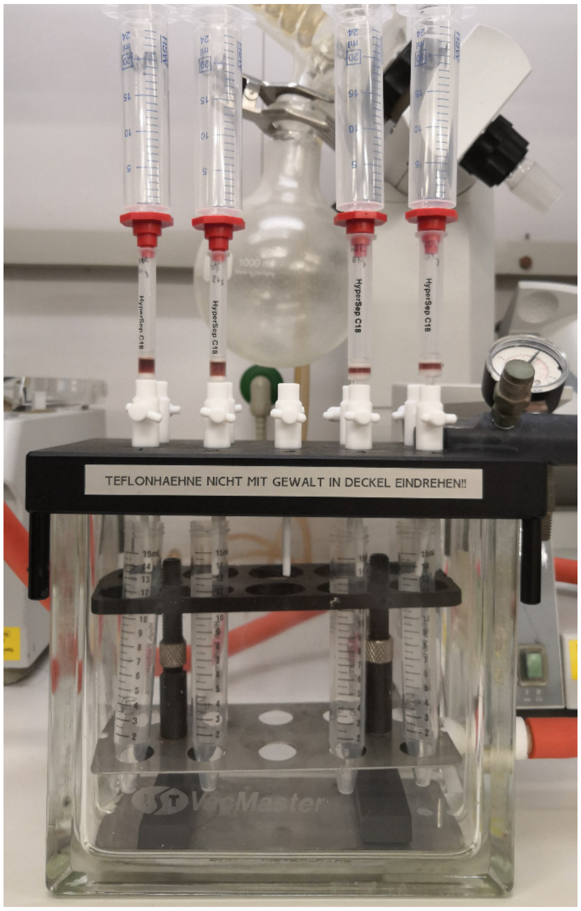


**Figure S1.** Experimental setup of siderophore attachment to $C_{18}$ solid-phase extraction columns. The setup shows the columns completely loaded with the cell-free culture supernatant.

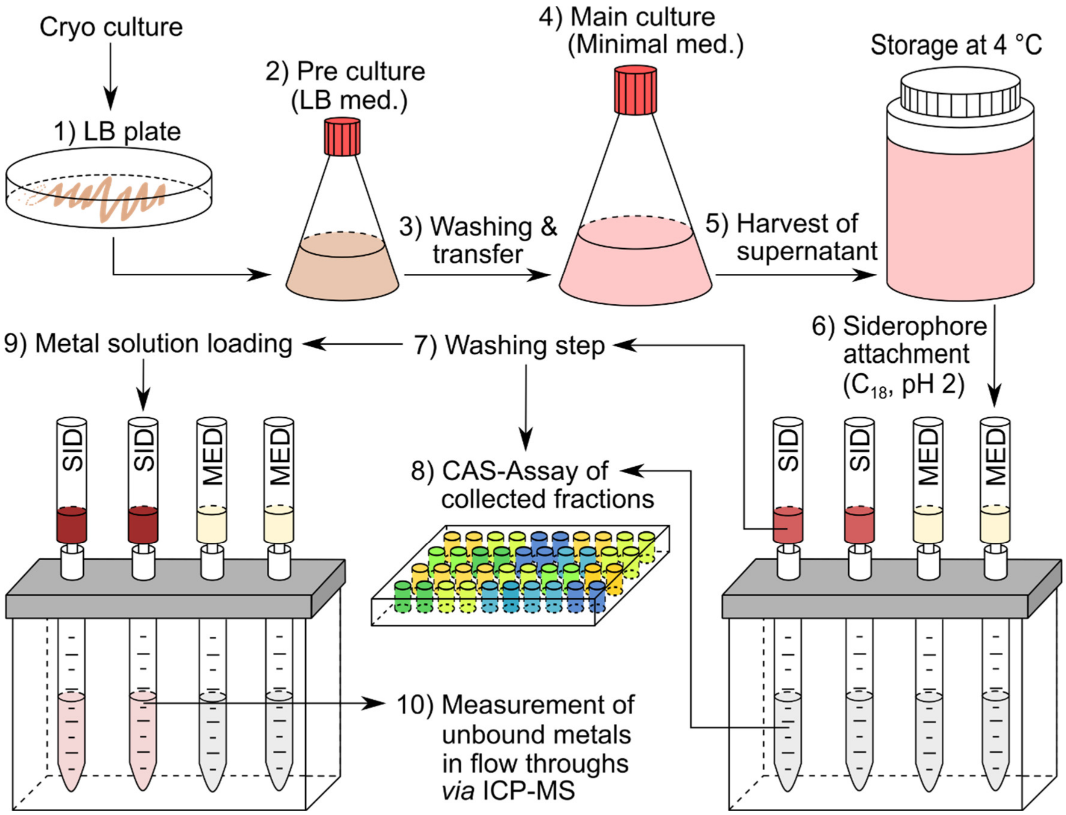


**Figure S2.** Workflow of cultivation, supernatant harvest, siderophore attachment and metal binding examination in bound state. Abbreviations: SID = siderophore; MED = medium.

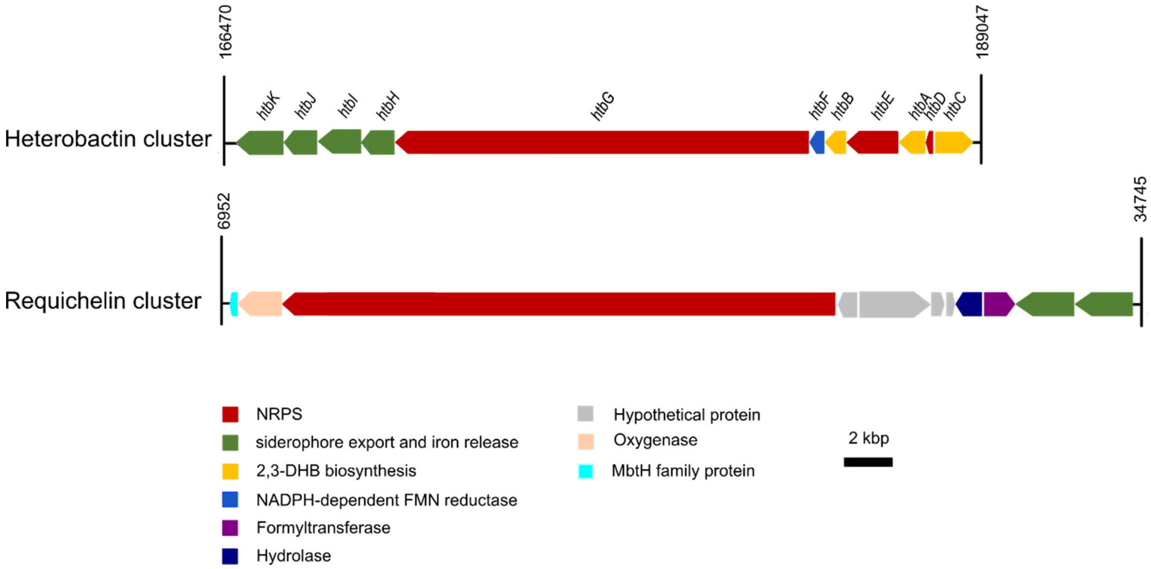


**Figure S3.** Organization of the biosynthesis gene clusters of *R. erythropolis* B7g.

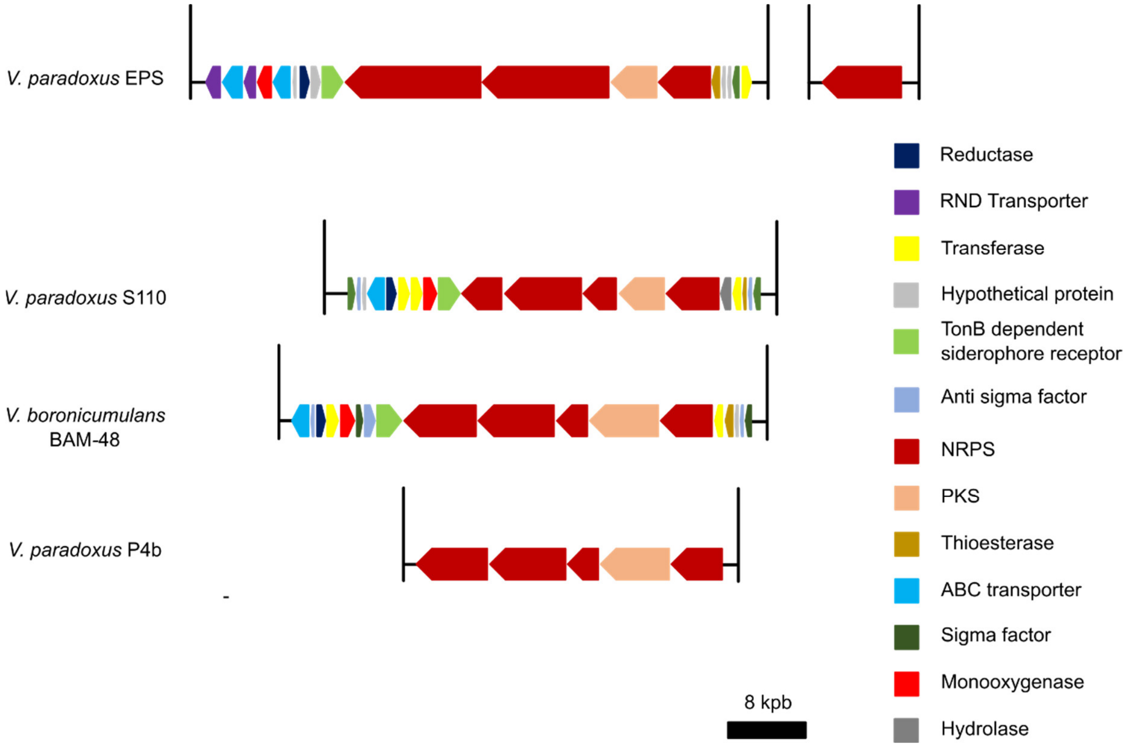


**Figure S4.** Organization of the biosynthesis gene cluster of *V. paradoxus* and other *Variovorax* sp.

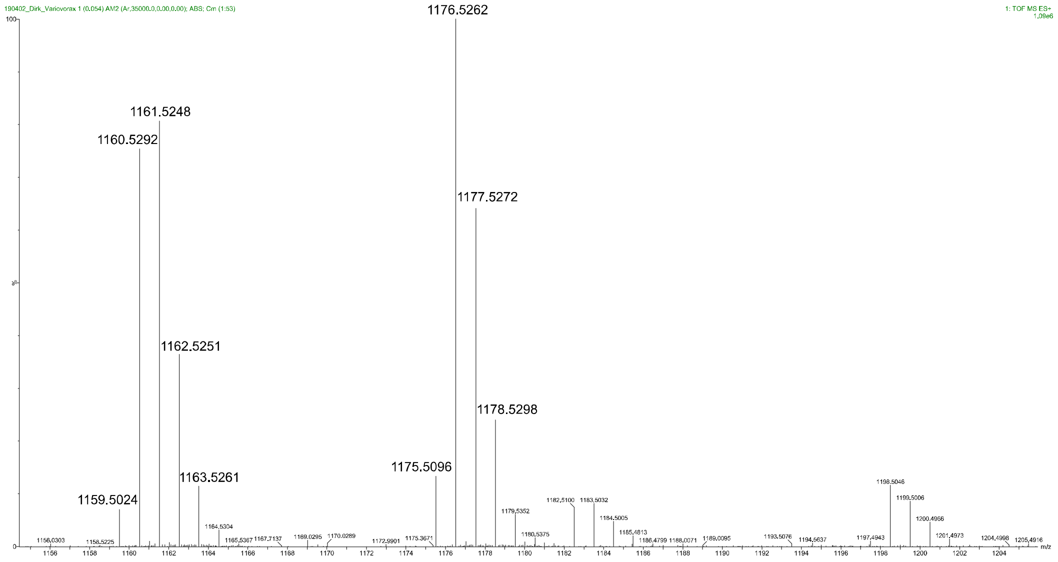


**Figure S5.** Mass spectrum of the siderophores of *Variovorax paradoxus* EPS. The spectrum was recorded on a mass spectrometer with an electrospray ionization source and a time of flight detector in $MS^E$ positive mode.

**Table S2.** Comparison of the genome sequences of *P. chlororaphis* DSM50083.

| Genome Feature | *Pseudomonas chlororaphis* DSM 50083 (This study) | *Pseudomonas chlororaphis* DSM 50083 |
|---|---|---|
| BioSample | SAMN16418570 | SAMN08359200 |
| Accession | JADCLH000000000 | CP027712 |
| Year of publication | 2020 | 2018 |
| Assembly date | 2017 | 2016 |
| Assembly method | Spades | HGAP |
| Status | draft | complete circular |
| Coverage | 407x | 178x |
| Seq. method | Illumina | PacBio |
| Sum of contig length (bp) | 6,771,389 | 8,808,187 |
| Contigs | 35 (> 3000 bp) | 1 |
| G + C content (%) | 63 | 63 |
| Protein coding genes | 6128 | 6224 |
| Average gene length (bp) | 978 | - |
| Coding percentage (%) | 89 | - |
| RNAs | 45 | 83 |

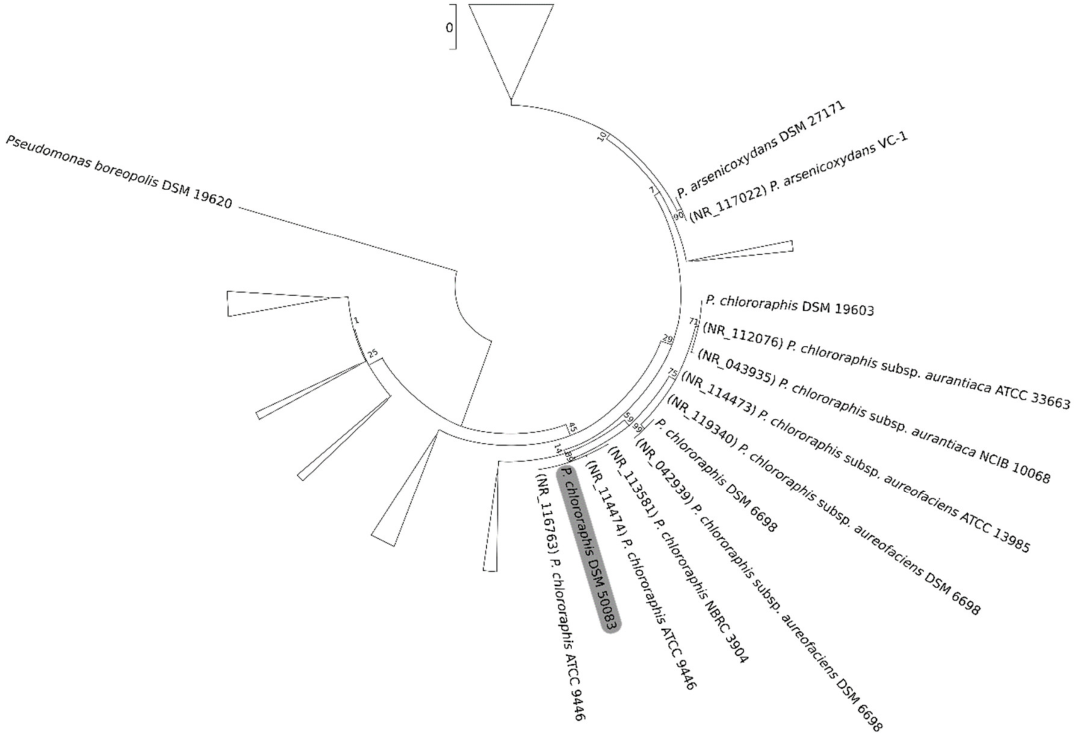


**Figure S6:** Phylogenetic tree of *Pseudomonas chlororaphis* relatives. The multiple sequence alignment on DNA level was done with 167 16S-sequences of *P. chlororaphis* DSM 50083 relatives by applying the ClustalW algorithm. Branches of distant relatives are condensed. The maximum likelihood tree was constructed by using MEGAX and bootstraps of 500 replicates [9]. Bootstrap values are indicated.

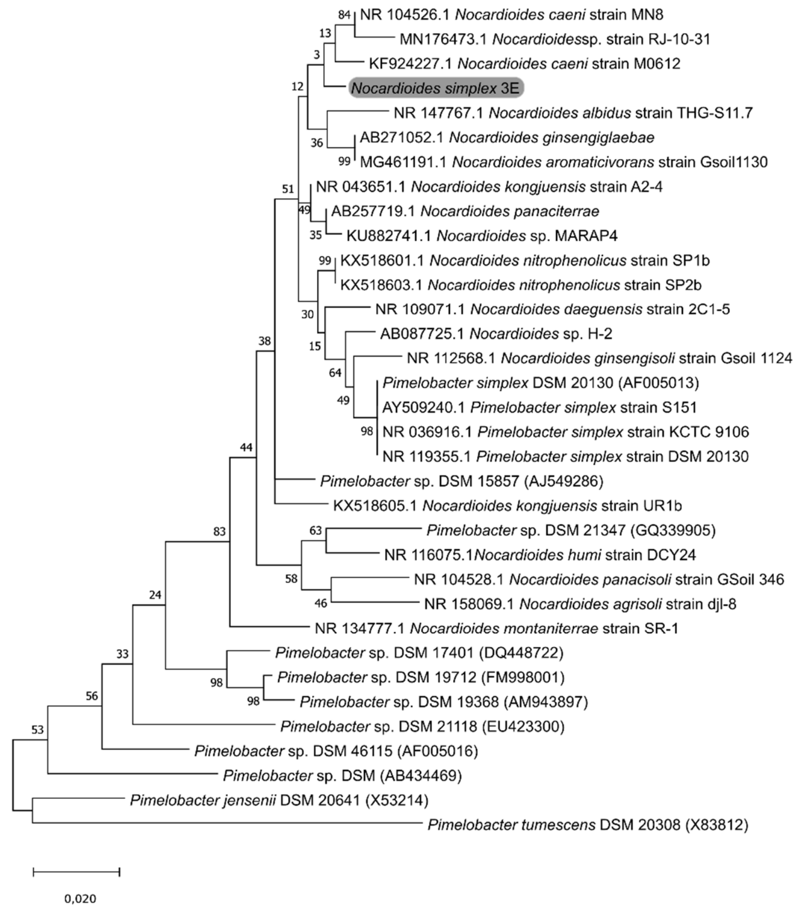


**Figure S7:** Phylogenetic tree of *Nocardioides simplex* relatives. The multiple sequence alignment on DNA level was done with 34 16S-sequences of *N. simplex* 3E relatives by applying the ClustalW algorithm. The maximum likelihood tree was constructed by using MEGAX and bootstraps of 500 replicates [9]. Bootstrap values are indicated.

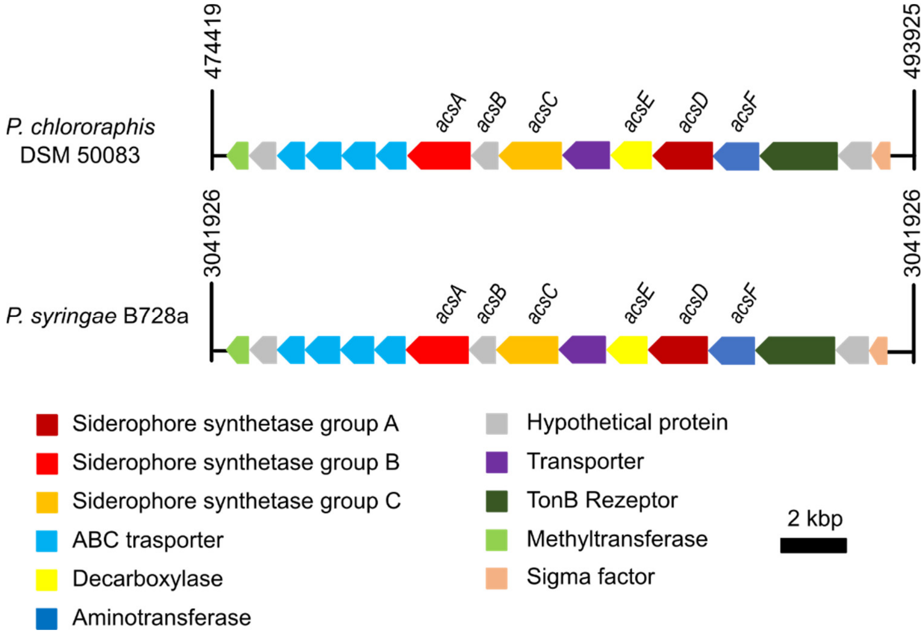


**Figure S8.** Organization of the achromobactin biosynthesis gene clusters in *P. chlororaphis* DSM 50083 and *P. syringae* B728a.

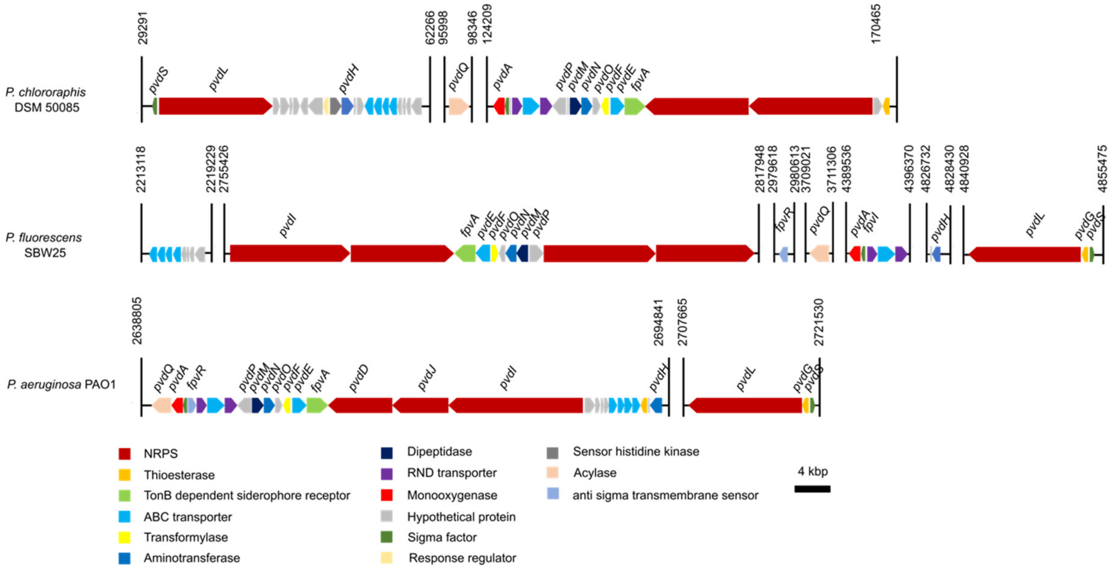


**Figure S9.** Organization of the pyoverdine biosynthesis gene cluster in *P. chlororaphis* DSM 50083, *P. aeruginosa* PAO1, and *P. fluorescens* SBW25.

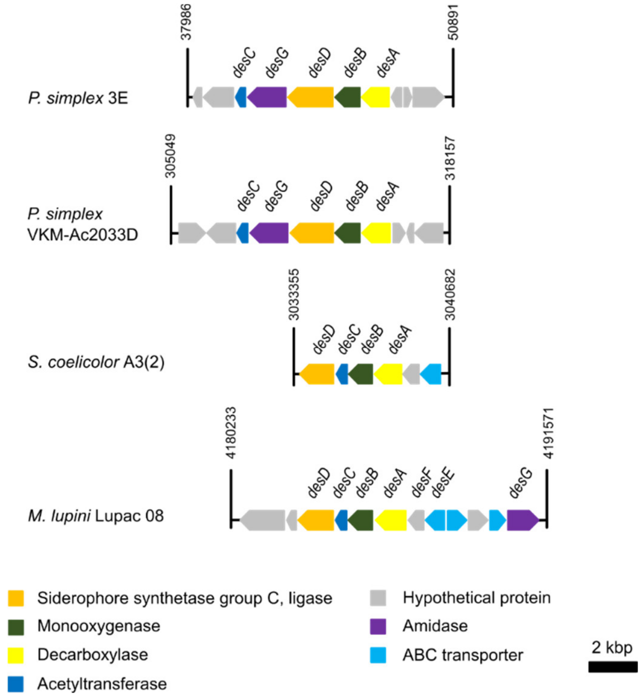


**Figure S10.** Organization of the biosynthesis gene cluster in *N. simplex* 3E and other strains containing desferrioxamine biosynthesis gene cluster.

**Table S3.** Optical density at inoculation and at stationary stage as reference for bacterial growth under different growth condition.

| | $OD_{600}$(inoculation) | $OD_{600}$(stationary phase) |
|---|---|---|
| ***P. chlororaphis* DSM 50083** | | |
| Glucose | | 0.98 ± 0.00 |
| Benzoate | | 0.68 ± 0.00 |
| Succinate | 0.48 ± 0.02 | 0.62 ± 0.05 |
| n-Hexadecane | | 0.394 ± 0.0 |
| ***R. erythropolis* B7g** | | |
| Glucose | | 1.25 ± 0.12 |
| n-Hexadecane | | 1.17 ± 0.22 |
| Glucose + n-Hexadecane | 0.30 ± 0.00 | n.d. |
| ***N. simplex* 3E** | | |
| Glucose | | 0.45 ± 0.04 |
| Succinate | | 0.32 ± 0.02 |
| Benzoate | 0.24 ± 0.00 | 0.35 ± 0.02 |
| n-Hexadecane | | 0.22 ± 0.03 |
| ***V. paradoxus* EPS** | | |
| Glucose | | 0.30 ± 0.02 |
| Succinate | 0.05 ± 0.00 | 0.19 ± 0.03 |
| Benzoate | | 0.18 ± 0.01 |
| n-Hexadecane | | 0.05 ± 0.00 |

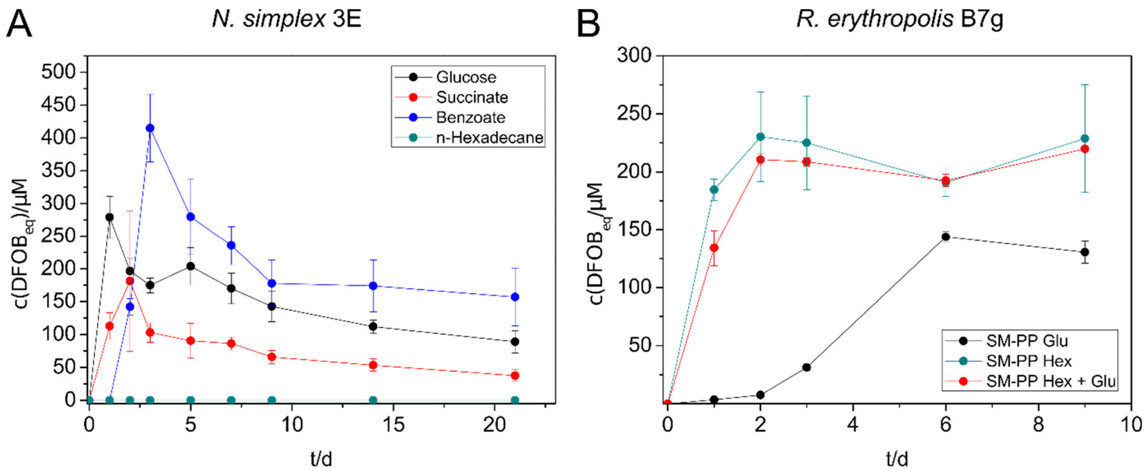


**Figure S11.** Siderophore production of *N. simplex* 3E (**A**) and *R. erythropolis* B7g (**B**) with different substrates and SM-PP medium. The siderophore production was monitored over time using the standard CAS assay. Experiments were done in triplicate (**A**) or duplicate (**B**).

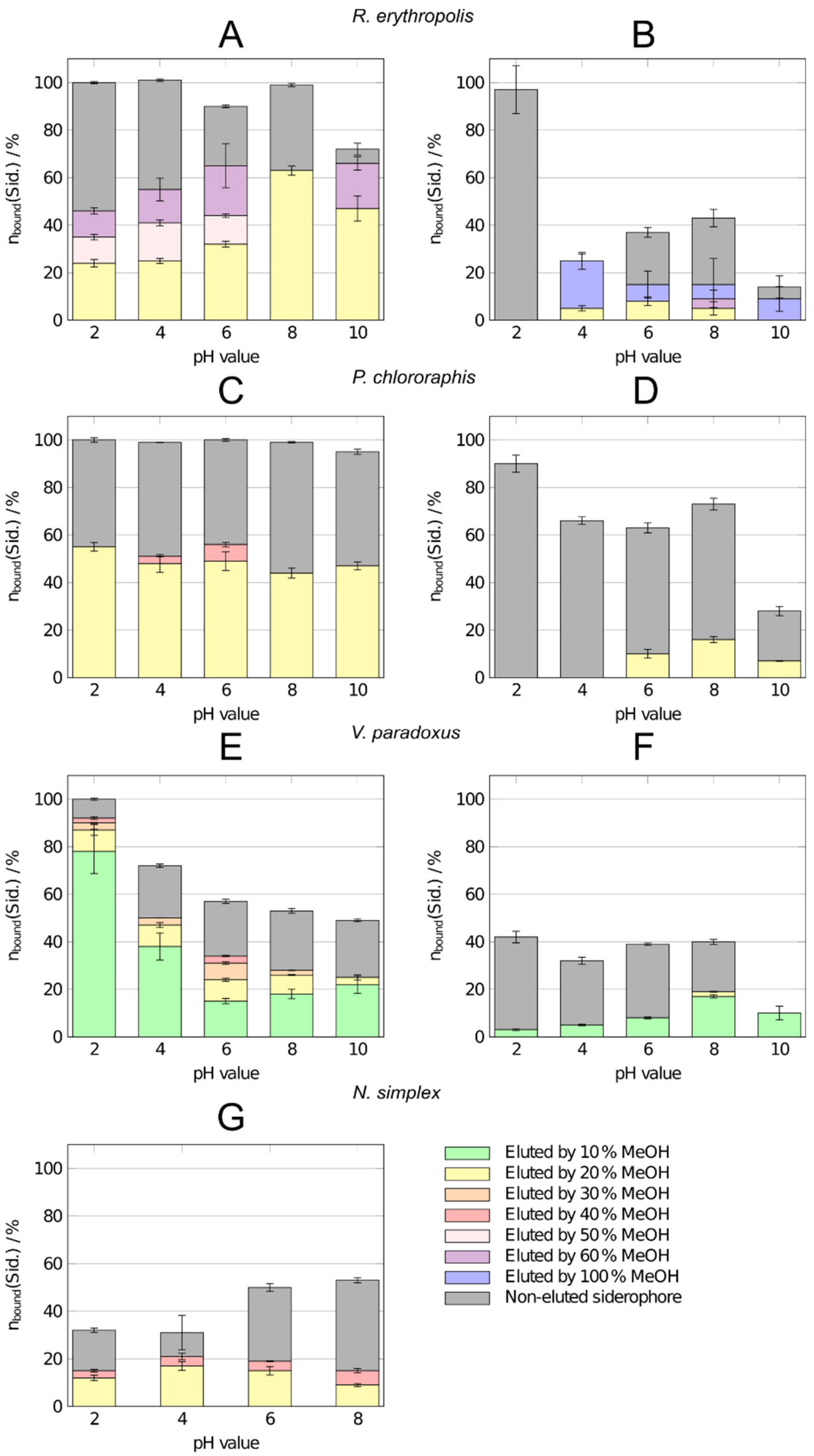


**Figure S12.** Siderophore attachment on solid-phase extraction columns. $C_{18}$ (A, C, E, G) or silica (B, D, F) SPE columns were loaded with siderophore-rich supernatant of *Rhodococcus erythropolis* B7g (A, B), *Pseudomonas chlororaphis* DSM 50083 (C, D), *Variovorax paradoxus* EPS (E, F) or *Nocardioides simplex* 3E (G) adjusted to various pH values. The experiment was carried out in duplicate. The loaded volume was 5 mL except for Sid-*Ns*3E (30 mL). The entire bar represents the % amount of $DFOB_{eq}$ bound to the solid phase. A washing step prior to elution was implemented in the case of Sid-*Ns*3E whereby about 3 % of the siderophore was washed out. The siderophore amount eluted by increasing methanol concentrations (10, 20, 30, 40, 50, 60, and 100 % MeOH) is represented by the colored parts of each bar. The grey portion reflects the siderophore quantity remaining on the column even after elution.

**Table 4.** Liquid chromatography gradient.

| Time [min] | % ACN + 0.1% FA |
|---|---|
| 0 | 98.0 |
| 2 | 98.0 |
| 9 | 0.5 |
| 11 | 0.5 |
| 13 | 98.0 |
| 14 | 98.0 |

**Table S5.** Siderophores detected in XAD extracts of *N. simplex* 3E. All siderophores were detected in three independent biological replicates. CCS gives the chemical cross section as determined in ion mobility. Given are significant figures with measurement imprecision represented in the last digit after the comma.

| Siderophore | Theoretical Mass $[M+H]^+$ | Observed Mass $[M+H]^+$ | Δppm | Retention Time [min] | CCS (Å$^2$) | Most Abundant Fragment Mass |
|---|---|---|---|---|---|---|
| Bisucaberin | 401.2395 | 401.2379 | 3.9 | 3.78 | 186.03 | 84.0796 |
| Desferrioxamine B | 561.3606 | 561.3595 | 2.1 | 3.53 | 224.13 | 201.1227 |
| Desferrioxamine D3 | 589.3556 | 589.3569 | 2.3 | 4.60 | 235.07 | 201.1228 |
| Desferrioxamine E | 601.3556 | 601.3541 | 2.5 | 4.19 | 225.16 | 201.1228 |

**Table S6.** Siderophores detected in XAD extracts of *R. erythropolis* B7g. All siderophores were detected in three independent biological replicates. CCS gives the chemical cross section as determined in ion mobility. Given are significant figures with measurement imprecision represented in the last digit after the comma.

| Siderophore | Theoretical mass $[M+H]^+$ | Observed mass $[M+H]^+$ | Δppm | Retention Time [min] | CCS (Å$^2$) | Most Abundant Fragment Mass |
|---|---|---|---|---|---|---|
| Heterobactin B | 438.1983 | 438.197 | -2.9 | 3.25 | 209.31 | 115.0859 |
| Apo-Heterobactin S2 | 694.2137 | 694.212 | -2.4 | 3.76 | 247.54 | 480.2199 |

**References**


1. Vogt, C.; Alfreider, A.; Lorbeer, H.; Hoffmann, D.; Wuensche, L.; Babel, W. Bioremediation of chlorobenzene-contaminated ground water in an in situ reactor mediated by hydrogen peroxide. *J. Contam. Hydrol.* **2004**, *68*, 121–141, doi:10.1016/S0169-7722(03)00092-5.
2. Gröning, J.A.D.; Kaschabek, S.R.; Schlömann, M.; Tischler, D. A mechanistic study on SMOB-ADP1: an NADH:flavin oxidoreductase of the two-component styrene monooxygenase of *Acinetobacter baylyi* ADP1. *Arch. Microbiol.* **2014**, *196*, 829–845, doi:10.1007/s00203-014-1022-y.
3. Kleeberg, S.B. *Isolation, Charakterisierung und Identifizierung von gram-positiven Bakterien mit der Fähigkeit den Umweltschadstoff Styrol abzubauen und Untersuchungen des Styrolabbauweges bei einem Isolat.*; Besondere Lernleistung Geschwister-Scholl-Gymnasium Nossen, Nossen, Germany, 2010.
4. Tischler, D.; Gröning, J.A.D.; Kaschabek, S.R.; Schlömann, M. One-component styrene monooxygenases: An evolutionary view on a rare class of flavoproteins. *Appl. Biochem. Biotechnol.* **2012**, *167*, 931–944, doi:10.1007/s12010-012-9659-y.
5. Dabrock, B.; Kesseler, M.; Averhoff, B.; Gottschalk, G. Identification and characterization of a transmissible linear plasmid from *Rhodococcus erythropolis* BD2 that encodes isopropylbenzene and trichloroethene catabolism. *Appl. Environ. Microbiol.* **1994**, *60*, 853–860, doi:10.1128/AEM.60.3.853-860.1994.
6. Oelschlägel, M.; Zimmerling, J.; Schlömann, M.; Tischler, D. Styrene oxide isomerase of *Sphingopyxis* sp. Kp5.2. *Microbiology* **2014**, *160*, 2481–2491, doi:10.1099/mic.0.080259-0.
7. Oelschlägel, M.; Rückert, C.; Kalinowski, J.; Schmidt, G.; Schlömann, M.; Tischler, D. *Sphingopyxis fribergensis* sp. nov., a soil bacterium with the ability to degrade styrene and phenylacetic acid. *Int. J. Syst. Evol. Microbiol.* **2015**, *65*, 3008–3015, doi:10.1099/ijs.0.000371.
8. Mehnert, M.; Retamal-Morales, G.; Schwabe, R.; Vater, S.; Heine, T.; Levicán, G.J.; Schlömann, M.; Tischler, D. Revisiting the Chrome Azurol S Assay for Various Metal Ions. *Solid State Phenom.* **2017**, *262*, 509–512, doi:10.4028/www.scientific.net/SSP.262.509.
9. Kumar, S.; Stecher, G.; Li, M.; Knyaz, C.; Tamura, K. MEGA X: Molecular evolutionary genetics analysis across computing platforms. *Mol. Biol. Evol.* **2018**, *35*, 1547–1549, doi:10.1093/molbev/msy096.
